# Predictability beyond accuracy: A correlation-based evaluation of survey forecasts of the Chilean exchange rate

**DOI:** 10.1371/journal.pone.0344095

**Published:** 2026-03-27

**Authors:** Pablo Pincheira, Lorenzo Reus, Andrea Bentancor, Martin Flores

**Affiliations:** 1 Escuela de Negocios, Universidad Adolfo Ibáñez, Santiago, Chile; 2 Facultad de Economía y Negocios, Universidad de Talca, Talca, Chile; State University of New York at Oswego, UNITED STATES OF AMERICA

## Abstract

Floating exchange rates are widely considered difficult—if not impossible—to predict. While traditional evaluations focus on out-of-sample accuracy measures such as Mean Squared Prediction Error (MSPE), recent literature argues that predictability is better understood as a form of dependence. Following this view, we assess the ability of Chile’s Survey of Professional Forecasters (SPF) to predict the Chilean peso (CLP) across multiple horizons. We find that SPF forecasts maintain stable and statistically significant predictive correlations with CLP returns, indicating meaningful predictability. However, forecast accuracy varies over time, mainly due to a persistent positive bias in the survey. We propose an adjustment aimed at removing this and other inefficiencies, which greatly improves accuracy, particularly at medium and long horizons. Finally, and contrary to common wisdom, we find that the most difficult benchmark to beat in the Chilean case is the random walk with drift—not the driftless random walk.

## 1. Introduction

In this paper, we evaluate the ability of the Survey of Professional Forecasters (SPF) in Chile to predict the Chilean exchange rate (CLP) at various horizons. As demonstrated by [1], the Driftless Random Walk (DRW) has proven to be a very difficult benchmark to outperform in out-of-sample evaluations within the exchange rate literature. Since then, a vast body of research has sought to explain why exchange rates exhibit near-random walk behavior [2] or why the DRW is so challenging [3]. Similarly, several studies have used new methods, tests and models, attempting to outperform the random walk. For example, [4] studies new predictors for several commodity-currencies, focusing on whether the prices of the commodities that dominate each country’s export basket can help explain and forecast their bilateral exchange rates. Using primarily daily data, the authors first examine whether these commodity prices deliver a strong out-of-sample fit for the corresponding exchange rates; an exercise in the spirit of [1]; though not a true forecasting evaluation because it relies on contemporaneous commodity prices. At the daily frequency, this out-of-sample fit is notably strong. Their analysis centers on the key export commodity associated with each exchange rate, namely oil for the Canadian dollar (CAD/USD), the Norwegian krone (NOK/USD), and the Australian dollar (AUD/USD); copper for the Chilean peso (CLP/USD); and gold for the South African rand (ZAR/USD). They then turn to traditional predictability tests, using lagged commodity prices in standard predictive regressions. In this setting, the evidence of forecasting power is considerably weaker: improvements relative to the DRW emerge only sporadically, in particular subsamples or at selected horizons. Forecast evaluation relies on Diebold–Mariano tests and [5] statistics, ensuring valid comparisons against nested benchmarks. The contribution of [4] is framed as an extension of the commodity-currency hypothesis developed in [6], where the authors documented significant predictive power from commodity-currency exchange rates toward commodity prices. The direction of analysis is effectively reversed by [4], asking whether commodity prices themselves can forecast exchange rates, and whether such predictive relationships survive rigorous out-of-sample testing. Overall, the study concludes that while contemporaneous commodity prices track high-frequency exchange-rate movements remarkably well, the lagged commodity prices offer only limited and fragile evidence of true out-of-sample predictability.

Another interesting article (See Ref. [7]) broadens the set of exchange rate models evaluated against the random walk, by introducing four new specifications: a real interest rate differential model incorporating shadow rates, a Taylor rule–based model, a sticky-price monetary model augmented with risk proxies, and an interest rate model that embeds yield-curve factors. Despite this expanded and more contemporary suite of fundamentals, the authors find that these newer models do not systematically outperform the older ones. Overall, while a handful of their results are noteworthy, they conclude that the long-standing question of exchange rate predictability remains largely unresolved.

More recently, [8] explores the usefulness of a new benchmark and a set of predictive variables for forecasting exchange rates, with particular attention to global risk measures that have gained prominence in recent years. Focusing on medium- and long-horizon forecasts, they assess whether these variables can systematically outperform the random walk model. Their analysis suggests that the apparent forecasting power of their proposed benchmark; the level of the exchange rate; and, by extension, of the global risk variables, is weak once sampling uncertainty is properly accounted for. In fact, their simulation-based evidence indicates that neither the new benchmark nor the global risk predictors provide strong evidence against the random walk model.

Although relatively recent studies, such as [9], have reported some improvements over the DRW, our reading of the literature suggests that this benchmark remains exceptionally difficult to beat in out-of-sample evaluations. Interestingly, [10] not only classifies the DRW as a strong benchmark but also points out that it is the most difficult to outperform. This observation is relevant to our work because, as we will see, in our case the Random Walk with drift (RW) tends to outperform the DRW at long horizons.

One strand of literature, not included in review [10], focuses on the evaluation of surveys of professional forecasters. See for instance, [11–13], and more recently, [14]. One interesting feature of this literature is that it covers floating exchange rates of both developed and emerging countries. In terms of predictive ability, results are mixed, with some surveys outperforming the DRW in the case of the Chilean Peso (e.g., [13]) and others being outperformed by the same benchmark (e.g., [14]).

The vast majority of the literature evaluating exchange rate predictability focuses on measures of forecast accuracy, like the popular Mean Squared Prediction Error (MSPE) and Mean Directional Accuracy (MDA). Yet, [15], makes a distinction between predictability and accuracy. They argue that predictability and forecast accuracy are two different yet related concepts. On the one hand, predictability is a notion of dependence between future and past events. A variable is predictable as long as its future is interconnected with some other variables in the present and in the past. This interpretation of predictability is also consistent with the views of [16,17]. On the other hand, forecast accuracy is just a measure of precision. The studies in [15,18] show that a zero forecast, which is totally independent of the target variable, may clearly display a lower MSPE than a forecast with a positive correlation of the target variable, yet displaying some degree of [19] inefficiency. This conflicting situation is labeled by the authors as the MSPE Paradox. Put differently, a forecast with a positive correlation with the target variable needs an additional requirement to be precise: it needs to be efficient as well, or mildly inefficient at most.

Building on these new concepts, this paper revisits the predictive performance of the SPF in the context of the Chilean Peso. The results in [13] previously demonstrated that the SPF outperformed the DRW across several horizons. However, their analysis did not address the presence of the MSPE Paradox, a key contribution of our study. Beyond this, it is essential to reassess the SPF’s predictive capabilities, given the significant global and domestic events of the past six years. These include the COVID-19 pandemic, the Russia-Ukraine war, and Chile’s 2019 social unrest, which triggered two pivotal referendums and led to a marked depreciation of the Chilean Peso.

**Fig 1:**
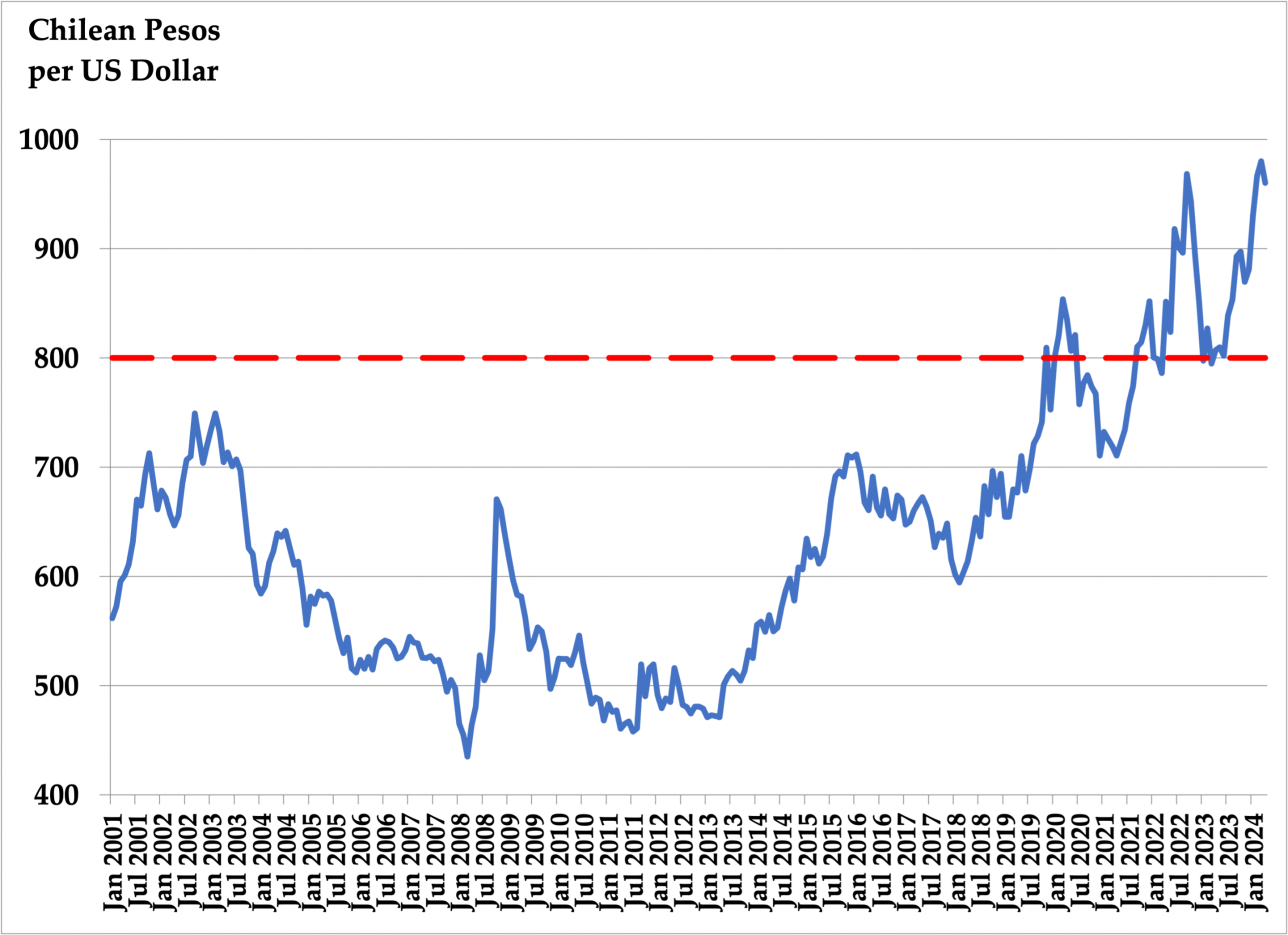
Monthly Chilean exchange rate relative to US dollar. Jan 2001–April 2024.

Fig 1 illustrates the Peso’s trajectory against the US Dollar throughout the SPF’s existence. While an upward trend emerged around 2012–2013, the Peso crossed the 800 Pesos per Dollar threshold shortly after the social unrest in October 2019. From January 2001 to September 2019, the Chilean currency averaged 588 Pesos per Dollar, compared to a much higher 824 Pesos per Dollar between October 2019 and April 2024. This substantial depreciation might have influenced the SPF’s predictive behavior, which we examine in detail in sections 3.2 and 3.3.

The potential discovery of strong predictors for the Chilean Peso is a topic of interest for both scholars and practitioners interested in exchange rate dynamics. Yet, as the Chilean Peso is also a commodity-currency, its potential predictability could also offer valuable insights regarding the ability to predict commodity prices, making it a topic of interest for a global audience. This relevance is emphasized by a growing body of research demonstrating the Peso’s ability to Granger-cause certain commodity prices, as shown in the seminal work of [6] and further supported by [20–22]. While our focus on the Chilean exchange rate may initially seem regionally specific, these findings suggest broader implications that resonate well beyond Chile.

As mentioned previously, [13] showed that the SPF consistently outperformed the DRW at multiple horizons when forecasting the Chilean Peso. However, closer scrutiny reveals that the traditional DRW, widely used as a benchmark, may not provide a fair comparison in this case due to differences in information. The DRW relies on end-of-month data to predict the exchange rate *h* periods ahead, whereas the SPF is typically released around the 10th day of each month. This means that the SPF benefits from approximately 10 additional days of market information, creating an inherent advantage over the DRW. As such, the superior performance of the SPF relative to the DRW, as reported by [13], may be partially attributable to this informational discrepancy rather than to the predictive strength of the SPF itself.

To address this issue, we propose a new benchmark: the “Driftless Random Walk Plus” (DRW+). Unlike the traditional DRW, the DRW+ uses exchange rate data from the day immediately preceding the SPF’s release, ensuring a more level playing field. This refinement, however, restricts the analysis to the period beginning in April 2012, when the Central Bank of Chile (CBCH) began disclosing the precise dates of SPF collection and release. Consequently, our study focuses on the April 2012–April 2024 period and further subdivides this interval into two segments: April 2012–May 2018 and June 2018–April 2024. While the first subsample aligns with the original analysis of [13], the second extends the evaluation into a period characterized by significant events, including Chile’s social unrest in 2019, the COVID-19 pandemic, and the Russia-Ukraine conflict, all of which may have had profound impacts on the Chilean Peso.

Our main findings indicate that: (1) Differing from [10], the Random Walk with drift (RW) consistently outperforms the Driftless Random Walk (DRW); (2) the enhanced “RW plus” (RW+) provides further improvements over the traditional RW; (3) the Survey of Professional Forecasters (SPF) exhibits mixed performance in terms of accuracy, achieving strong results in the first half of the sample period but being outperformed by our naïve benchmarks in the second half; and (4) the SPF maintains a positive and statistically significant correlation with Chilean Peso returns across short, medium, and long horizons throughout the entire sample. These findings suggest the presence of the MSPE Paradox and confirm that the SPF does predict the Chilean Peso, though with time-varying accuracy. This variability is primarily driven by a persistent and positive bias in the SPF forecasts. To address this issue, we propose an Adjusted forecast, which significantly outperforms the original SPF, particularly at medium and long horizons.

The structure of this paper is as follows: Section 2 outlines the dataset used for the analysis. In Section 3.1, we compare the predictive performance of four different benchmarks: DRW, DRW + , RW, and RW + . Sections 3.2–3.4 focus on the evaluation of the SPF’s predictive accuracy and on its efficiency. Finally, we conclude in Section 4.

## 2. Materials and methods

We utilize monthly data spanning from April 2012 to April 2024. This period is the only timeframe during which the Central Bank of Chile (CBCH) publicly reports the release dates of the Survey of Professional Forecasters (SPF).

Our primary data source is the monthly SPF published by the CBCH. The survey is typically conducted during the first two weeks of each month, with results released to the public the day after collection. Throughout the sample period, the release date fluctuates between the 9th and 13th of each month. The survey targets economists, consultants, and executives from the financial sector. The CBCH reports the median values and the 10th and 90th percentiles for each forecast. Exchange rate forecasts are provided for three different horizons: 2 months (SPF2), 11 months (SPF11), and 23 months (SPF23) ahead. For a more detailed methodology of the survey, refer to [23]. Data were accessed for research purposes on June 10 2024. The authors did not have access to information that could identify individual participants during or after data collection.

For exchange rate data, we extract the daily closing price of the Chilean Peso (CLP) from Bloomberg. These data are converted to monthly frequencies by sampling from the last day of each month. We also sample the closing price from the day before the survey is released, which is simply denoted as CLP + .

**Fig 2:**
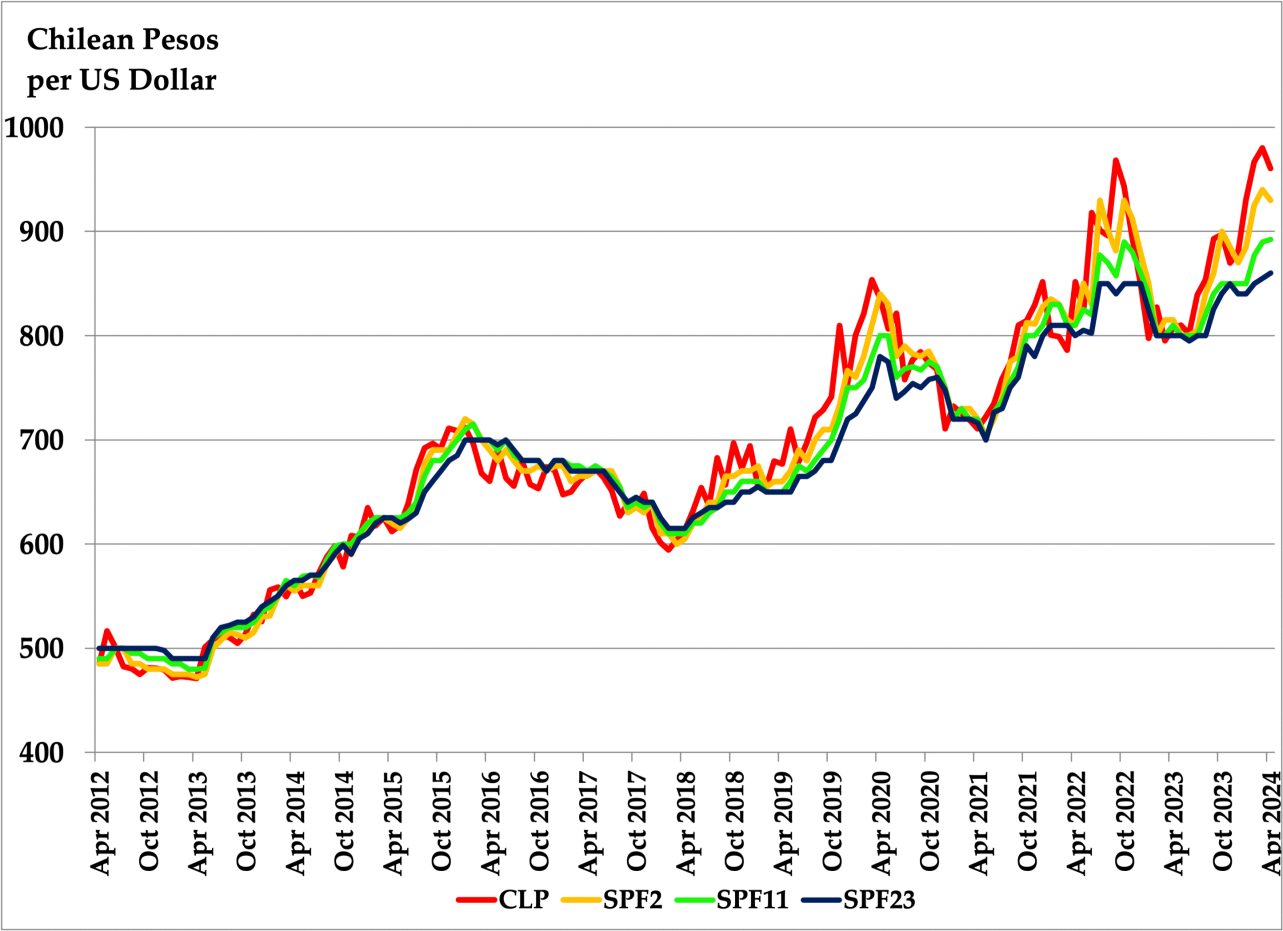
Chilean exchange rate relative to US dollar and SPF forecasts. April 2012 – April 2024.

Fig 2 illustrates the Chilean Peso alongside the three SPF forecasts (SPF2, SPF11, and SPF23) for the entire sample period. This figure reveals two key observations: first, the Chilean Peso has followed a clear upward trend throughout the period; second, although all three SPF forecasts track the CLP closely, they tend to underestimate the exchange rate in the second half of the sample.

To assess predictive performance, we define three evaluation windows: (1) the full sample period from April 2012 to April 2024, (2) the first subsample from April 2012 to May 2018, and (3) the second subsample from June 2018 to April 2024.

## 3. Results

### 3.1. Which is the toughest benchmark?

In this subsection we explore a fairly simple question: which is the right model to use as a benchmark? According to the review in [10], the toughest benchmark to beat in the exchange rate literature is the DRW. Yet, as mentioned in the introduction, when compared to a survey that has an informational advantage of about 10 days, we rather expect this survey to outperform the DRW.

We are now in need of introducing some notation. Let ${\text{S}}_{\text{t}}$ denote the nominal exchange rate at time *t*, defined as the number of Chilean pesos required to purchase one U.S. dollar. Specifically, ${\text{S}}_{\text{t}}$ represents the closing price of the Chilean peso on the final trading day of month *t*. For example, for January 2024, ${\text{S}}_{\text{t}}$ corresponds to the closing price recorded on Wednesday, January 31.

We will use lower-case letters to denote the natural logarithm of a given variable, so: $s_{t}\equiv \text{ln}\left(S_{t}\right).$ Let us define the *h*-period return of the Chilean peso as $r_{t,t+h}=s_{t+h}-s_{t}$. We are interested in forecasts of this variable at various horizons denoted by *h*. We consider *h* = 1, 2, 3, 6, 9, 11, 12, 18, 24 months (We focus on the horizons h = 1, 2, 3, 6, 11, 12, 18 and 24 months for three reasons. First, this set enables a direct comparison with [13], who consider the same horizons in their analysis. Second, the Central Bank of Chile requests forecasts 2, 11, and 23 months ahead in its survey, which we interpret as reference points for short-, medium-, and long-term expectations. We therefore evaluate nearby horizons: 1–3 months, 9–12 months, and 18–24 months. Third, the Bank’s monetary policy horizon, approximately 18–24 months, makes these longer horizons particularly relevant for policy considerations.).

A RW model for $s_{t}$ is simply given by


$$s_{t+\text{1}}=\alpha +s_{t}+\varepsilon_{t}$$ (1)

where $\varepsilon_{t}$ is a white noise process and α is a constant representing the systematic drift in the evolution of the exchange rate (A white noise $\varepsilon_{t}$ is a stationary time-series process satisfying the following three conditions: $E\left(\varepsilon_{t}\right)=0\text{\textbackslash{} \textbackslash{} for\textbackslash{} all\textbackslash{} t}$, $\text{Var}\left(\epsilon_{\text{t}}\right)=\sigma^{\text{2}}>0\text{ for all t}$, and finally $E(\varepsilon_{t}\;\varepsilon_{s})=0$ = 0 for all t, s such that t ≠ s.). In a random walk with drift, α captures the average expected change in $s_{t}$ that is not due to random shocks. This parameter governs the long-run direction of the series: a positive drift implies a persistent tendency for the exchange rate to depreciate over time, while a negative drift implies the opposite. Allowing the possibility of a non-zero drift matters because it enables the model to capture persistent trends; such as inflation differentials, risk premia or structural forces; that would otherwise be absorbed by the error term. When α is exactly zero, expression (1) defines a DRW model for $s_{t}.$ Using model (1), the optimal linear forecast for $r_{t,t+h}$ is given by $\overset{f}{\underset{t}{r}}(h)=\;\alpha h$. Again, when α=0 this forecast becomes $\overset{f}{\underset{t}{r}}(h)=\;\text{0}$ for all forecasting horizon *h*. We will introduce the following notation to clearly identify these different forecasts.


$$\overset{RW}{\underset{t}{r}}(h)=\;\alpha h$$

$$\overset{DRW}{\underset{t}{r}}(h)=\;\text{0}$$

Coming back to the previously mentioned informational advantage of the SPF relative to a RW, it seems natural to consider the following alternative target variable for the Chilean peso:


$$r_{t^{+},t+h}=s_{t+h}-s_{t^{+}}$$

We use the subscript “$t^{+}$” to explicitly remark that the respondents of the survey provide their forecasts approximately on the 10th day of month “*t+1*”. In other words, we have the following inequality: *t* < $t^{+}$< t + 1. For this new target variable $r_{t^{+},t+h},$ we propose two natural extensions of the RW and DRW benchmarks defined previously. These extensions are denoted by RW+ and DRW+ and provide the following forecasts for $r_{t^{+},t+h}$:


$$\overset{RW+}{\underset{t^{+}}{r}}(h)=\overset{+}{\underset{h}{\alpha }}$$

$$\;\overset{DRW+}{\underset{t^{+}}{r}}(h)=\text{0}$$

Here $\overset{+}{\underset{h}{\alpha }}$ is just a constant depending on the reference point “$t^{+}$” and the forecast horizon *h*. These new benchmarks use the closing price of the Chilean peso from the day before the survey is released $\left(s_{t^{+}}\right)$ as the initial exchange rate. The logic for changing the reference point is that an investor who reads the survey will be interested in the return of the Chilean peso from the reference point “$t^{+}$” onward, as the information between *t* and “$t^{+}$” is already known at time “$t^{+}$”.

Notice that the four benchmark forecasts $\overset{RW}{\underset{t}{r}}(h),\;\overset{DRW}{\underset{t}{\;r}}(h)$, $\overset{RW+}{\underset{t^{+}}{r}}(h)\text{ and }\overset{DRW+}{\underset{t^{+}}{r}}(h)$ can be used to generate predictions for $s_{t+h}$. They basically are


$$\overset{RW}{\underset{t}{s}}(h)=\;\alpha h+s_{t}$$

$$\overset{DRW}{\underset{t}{s}}(h)=\;s_{t}$$

$$\overset{RW+}{\underset{t^{+}}{s}}(h)=\;\overset{+}{\underset{h}{\alpha }}+s_{t^{+}}$$

$$\overset{DRW+}{\underset{t^{+}}{s}}(h)=\;s_{t^{+}}$$

In this sense they can be considered as forecasts for the same target variable. From that point of view it is worth to explore and compare their accuracy. To make this point clearer, notice that when forecasting either $r_{t,t+h}$ or $s_{t+h}$, forecast errors are the same. For returns, forecast errors are defined as


$$\overset{RW}{\underset{t}{e}}(h)=\;r_{t,t+h}-\overset{RW}{\underset{t}{r}}(h)={\text{s}}_{\text{t}+\text{h}}-{\text{s}}_{\text{t}}-\alpha h$$

$$\overset{DRW}{\underset{t}{e}}(h)=\;r_{t,t+h}-\overset{DRW}{\underset{t}{r}}(h)={\text{s}}_{\text{t}+\text{h}}-{\text{s}}_{\text{t}}$$

For $s_{t+h}$, forecast errors are defined as


$$\overset{RW}{\underset{t}{u}}(h)=\;{\text{s}}_{\text{t}+\text{h}}-\overset{RW}{\underset{t}{s}}(h)={\text{s}}_{\text{t}+\text{h}}-{\text{s}}_{\text{t}}-\alpha h$$

$$\overset{DRW}{\underset{t}{u}}(h)=\;{\text{s}}_{\text{t}+\text{h}}-\overset{DRW}{\underset{t}{s}}(h)={\text{s}}_{\text{t}+\text{h}}-{\text{s}}_{\text{t}}$$

When forecasting from *“$t^{+}$”* we observe the same situation. For returns, forecast errors are defined as


$$\overset{RW}{\underset{t^{+}}{e}}(h)=\;{\text{r}}_{t^{+},\text{t}+\text{h}}-\overset{RW+}{\underset{t^{+}}{r}}(h)={\text{s}}_{\text{t}+\text{h}}-{\text{s}}_{t^{+}}-\overset{+}{\underset{h}{\alpha }}$$

$$\overset{DRW}{\underset{t^{+}}{e}}(h)=\;{\text{r}}_{t^{+},\text{t}+\text{h}}-\overset{DRW+}{\underset{t^{+}}{r}}(h)={\text{s}}_{\text{t}+\text{h}}-{\text{s}}_{t^{+}}$$

Yet, for $s_{t+h},$ forecast errors are defined as


$$\overset{RW}{\underset{t^{+}}{u}}(h)=\;{\text{s}}_{\text{t}+\text{h}}-\overset{RW}{\underset{t^{+}}{s}}(h)={\text{s}}_{\text{t}+\text{h}}-{\text{s}}_{t^{+}}-\overset{+}{\underset{h}{\alpha }}$$

$$\overset{DRW}{\underset{t^{+}}{u}}(h)=\;{\text{s}}_{\text{t}+\text{h}}-\overset{DRW}{\underset{t^{+}}{s}}(h)={\text{s}}_{\text{t}+\text{h}}-{\text{s}}_{t^{+}}$$

We have shown that when forecasting either returns $r_{t,t+h}$ or log-levels $s_{t+h}$ we obtain the same forecast errors. Notice, however, that when changing the reference point from *t* to *“$t^{+}$”*, these errors naturally change. As *“$t^{+}$”* is closer to *t + 1,* we expect forecast errors indexed by *“$t^{+}$”* to display a lower MSPE. Furthermore, findings of significant differences between forecasts using different reference points, (*t* vs *“$t^{+}$”*) suggest that the good performance of the SPF relative to the DRW reported in [13] might be importantly affected by the informational advantage of the survey.

Probably the most common metric used to gauge forecast accuracy is the Mean Squared Prediction Error (MSPE), defined as the expected value of the squared forecast errors. For instance, when forecasting h-steps with the DRW model, the corresponding MSPE is


$$\overset{DRW}{\underset{h}{MSPE}}=E\left[{\left(\overset{DRW}{\underset{t}{e}}(h)\right)}^{\text{2}}\right]$$

Given a sample of P(h) available h-step-ahead forecast errors, the MSPE is typically estimated as


$$\overset{DRW}{\underset{h}{\hat{MSPE}}}=\frac{\text{1}}{\text{P}(\text{h})}\sum_{t=\text{1}}^{\text{P}(\text{h})}{\left[\overset{DRW}{\underset{t}{e}}(h)\right]}^{\text{2}}$$

It is also common to report the square root of the MSPE as an alternative accuracy measure. This is denoted RMSPE. To simplify notation, we will use the term MSPE to refer indistinctly to both the population quantity and its sample counterpart.

Table 1 reports Root MSPE for the DRW and DRW+ in our full sample and in the two subsample periods of interest: April 2012-May 2018 and June 2018-April 2024. Table 1 also reports RMSPE ratios. A number lower than 1 favors the DRW+ benchmark. The table clearly shows that the DRW+ is more accurate than the traditional DRW at every single horizon, with only one exception that occurs when forecasting 24 months ahead in Panel 2. In this particular case both benchmarks display almost identical accuracy, with a tiny edge in favor of the DRW. Aside from this case, in all three Panels the DRW+ shows higher accuracy relative to the DRW, especially at short horizons of 1–6 months. At longer horizons, the informational advantage of these approximately 10 extra days of market information decreases considerable, but at shorter horizons the superior predictive accuracy of the DRW+ over the traditional DRW is substantial, with RMSPE Ratios as low as 0.82.

**Table 1:** RMSPE Comparison between DRW+ and DRW.

Panel 1: Full Sample Period									
Horizon h (months)	1	2	3	6	9	11	12	18	24
**RMSPE Ratio**	0.83	0.88	0.89	0.95	0.98	0.99	0.99	0.99	0.99
**DRW+**	2.88	3.87	4.96	7.60	9.70	10.48	10.91	13.16	15.74
**DRW**	3.46	4.37	5.57	7.99	9.90	10.57	11.01	13.27	15.87
**Panel 2: April 2012-May 2018**									
**Horizon h (months)**	**1**	**2**	**3**	**6**	**9**	**11**	**12**	**18**	**24**
**RMSPE Ratio**	0.86	0.94	0.97	0.99	0.98	0.99	0.99	0.99	1.00
**DRW+**	2.30	3.40	4.25	6.04	8.26	9.49	10.13	14.29	18.63
**DRW**	2.67	3.61	4.37	6.09	8.40	9.60	10.21	14.38	18.61
**Panel 3: June 2018-April 2024**									
**Horizon h (months)**	**1**	**2**	**3**	**6**	**9**	**11**	**12**	**18**	**24**
**RMSPE Ratio**	0.82	0.85	0.85	0.94	0.98	0.99	0.99	0.99	0.97
**DRW+**	3.38	4.29	5.59	8.86	10.88	11.30	11.56	12.18	13.27
**DRW**	4.11	5.03	6.55	9.46	11.10	11.36	11.67	12.33	13.61

Table 1 shows the accuracy of DRW and DRW+ benchmarks when forecasting Chilean Peso returns at several horizons and in different samples. In particular, Table 1 displays the RMSPE of each benchmark and the RMSPE ratio between them. Ratios lower than 1 favors the DRW+ benchmark.

Table 2 is akin to Table 1, but comparing the RW+ with the RW. These two benchmarks require estimation of the $\overset{+}{\underset{h}{\alpha }}$ and α parameters respectively. We estimate them with Ordinary Least Squares (OLS) in rolling windows of 74 observations, which basically corresponds to our first subsample of interest. Consequently, when using either the RW+ or RW we only show results for the second subsample, from June 2018 until April 2024.

**Table 2:** RMSPE Comparison: RW+ vs RW.

June 2018 – April 2024									
Forecast horizon h (months)	1	2	3	6	9	11	12	18	24
**RMSPE Ratio**	0.83	0.86	0.86	0.94	0.98	0.99	0.99	0.99	0.96
**RW+**	3.39	4.25	5.49	8.56	10.11	10.17	10.35	10.71	12.77
**RW**	4.10	4.96	6.42	9.13	10.35	10.29	10.50	10.79	13.31

Table 2 shows the accuracy of RW and RW+ benchmarks when forecasting Chilean Peso returns at several horizons. In particular, Table 1 displays the RMSPE of each benchmark and the RMSPE ratio between them. Ratios lower than 1 favors the RW+ benchmark.

Results in Table 2 confirm the findings from Table 1, highlighting that the informational advantage of the RW+ model translates into greater forecast accuracy, particularly at shorter horizons. According to the review of [10], the DRW is the toughest benchmark to beat in the exchange rate literature. Nevertheless, Fig 1 and 2 show a clear upward trend in the Chilean Peso during our sample period. This is indication that a forecast like $\overset{RW}{\underset{t}{r}}(h)$ or $\overset{RW+}{\underset{t^{+}}{r}}(h)\;$ might be competitive as well.

Tables 3 and 4 next show RMSPE comparing both benchmarks in the traditional version (RW vs DRW in Table 3) and in the “plus” version (RW+ vs DRW+ in Table 4). Table 3 clearly shows that the RW is more accurate than the traditional DRW at every single horizon. RMSPE ratios have a U shape as a function of the forecast horizon, reaching a minimum of 0.88 when forecasting 18 months ahead. This is indication that the explicit inclusion of a linear trend is helpful when forecasting at medium horizons. Table 4 provides a similar picture. With the only exception of h = 1, the RW+ outperforms the DRW when *h* is greater or equal than 2, reaching a maximum difference when h = 18.

**Table 3:** RMSPE Comparison: RW vs DRW. June 2018 – April 2024.

Forecast horizon h (months)	1	2	3	6	9	11	12	18	24
**RMSPE Ratio**	1.00	0.98	0.98	0.97	0.93	0.91	0.90	0.88	0.98
**RW**	4.10	4.96	6.42	9.13	10.35	10.29	10.50	10.79	13.31
**DRW**	4.11	5.03	6.55	9.46	11.10	11.36	11.67	12.33	13.61

Table 3 shows the accuracy of RW and DRW benchmarks when forecasting Chilean Peso returns at several horizons. In particular, Table 1 displays the RMSPE of each benchmark and the RMSPE ratio between them. Ratios lower than 1 favors the RW benchmark.

**Table 4:** RMSPE Comparison: RW+ vs DRW+June 2018 – April 2024.

Forecast horizon h (months)	1	2	3	6	9	11	12	18	24
**RMSPE Ratio**	1.00	0.99	0.98	0.97	0.93	0.90	0.90	0.88	0.96
**RW+**	3.39	4.25	5.49	8.56	10.11	10.17	10.35	10.71	12.77
**DRW+**	3.38	4.29	5.59	8.86	10.88	11.30	11.56	12.18	13.27

Table 4 shows the accuracy of RW+ and DRW+ benchmarks when forecasting Chilean Peso returns at several horizons. In particular, Table 4 displays the RMSPE of each benchmark and the RMSPE ratio between them. Ratios lower than 1 favors the RW+ benchmark.

We finish this section with Table 5, which compares RMSPE of our less competitive benchmark (the DRW) with the most competitive benchmark (the RW+). Differences between these two competitors are sizable and statistically significant at short and medium horizons. This can be seen in the last row of Table 5, where we show traditional t-statistics of the Diebold-Mariano-West test [24–25] (Henceforth DMW test). This test evaluates the null of equal MSPE between these two benchmarks. As we can see, the null is rejected in favor of the RW+.

**Table 5:** RMSPE Comparison: RW+ vs DRW. June 2018 – April 2024.

Forecast horizon (months)	1	2	3	6	9	11	12	18	24
**RMSPE Ratio**	0.82	0.84	0.84	0.90	0.91	0.89	0.89	0.87	0.94
**RW+**	3.39	4.25	5.49	8.56	10.11	10.17	10.35	10.71	12.77
**DRW**	4.11	5.03	6.55	9.46	11.10	11.36	11.67	12.33	13.61
**DMW**	**3.07**	**4.30**	**3.48**	**1.97**	1.23	1.23	1.23	1.06	0.34

Table 5 shows the accuracy of the RW+ and DRW benchmarks when forecasting Chilean Peso returns at several horizons. In particular, Table 5 displays the RMSPE of each benchmark and the RMSPE ratio between both of them. Figures lower than 1 favors the RW+ . The last row in Table 5 shows traditional t-statistics of a DMW test evaluating the null hypothesis of equality in MSPE between both benchmarks. Positive values of this test favors the RW+ . Entries in bold denote statistically significant results at the 5% significance level. The DMW test is computed using HAC standard errors according to [26–27].

The fact that the RW performs better than the DRW in our setting may appear to contradict the conventional view—summarized in [10]—that the driftless random walk is the most difficult benchmark to beat. A key explanation is purely statistical: in many of the currency pairs analyzed in earlier studies, such as that by [8], the estimated drift is economically small or statistically indistinguishable from zero, making the DRW an appropriate benchmark. In contrast, in samples where the exchange rate exhibits a statistically meaningful drift—as in our case—the zero-drift restriction imposed by the DRW becomes inconsistent with the data. In such settings, the RW or RW+ naturally tends to perform better in forecast comparisons.

In the next section we compare the predictive performance of the SPF relative to our toughest benchmarks: the DRW+ when evaluating the whole sample and the RW+ when focusing on our more recent subsample of interest.

### 3.2. SPF forecast evaluation

In this subsection, we evaluate the ability of the SPF to predict CLP returns at several forecast horizons *h*, with *h* = 1, 2, 3, 6, 9, 11, 12, 18 and 24 months ahead. Our evaluation employs three types of analyses: traditional MSPE comparisons, MDA comparisons, and a novel yet straightforward approach proposed by [28], which aims to identify the forecast most strongly correlated with the target variable.

We evaluate differences in MSPE relative to the two most competitive benchmarks identified in Section 3.1: the DRW+ and the RW+ . When evaluating MDA we use a “pure luck” benchmark, which simply assumes that it is possible to correctly forecast the future direction of change with at least 50% of success. Finally, we use a correlation analysis to determine, among other aspects, whether the correlation of the SPF with the target variable is both positive and statistically significant, and to examine whether the MSPE Paradox arises in a meaningful way.

#### 3.2.1. MSPE forecast evaluation.

In this subsection we focus on the accuracy of the SPF when predicting Chilean Peso returns defined as


$$r_{t^{+},t+h}=s_{t+h}-s_{t^{+}}$$

We consider the following forecast coming from the SPF:


$$\overset{SPF}{\underset{t^{+}}{r}}=\overset{SPF}{\underset{t^{+}}{s}}-s_{t^{+}}\text{ for all h}$$ (2)

where $\overset{SPF}{\underset{t^{+}}{s}}\equiv ln\left(\overset{SPF}{\underset{t^{+}}{S}}\right)$ and $\overset{SPF}{\underset{t^{+}}{S}}$ is the forecast of the nominal exchange rate made at time $t^{+}$ coming from the survey. The corresponding forecast errors are given by


$$\overset{SPF}{\underset{t^{+}}{e}}(h)=s_{t+h}-\overset{SPF}{\underset{t^{+}}{s}}$$

Let us recall that we treat SPF2, SPF11, and SPF23 as distinct forecasts for $r_{t^{+},t+h}$. So, in what follows we will evaluate $\overset{SPF\text{2}}{\underset{t^{+}}{r}},\;\overset{SPF\text{1}\text{1}}{\underset{t^{+}}{\;r}}$ and $\overset{SPF\text{2}\text{3}}{\underset{t^{+}}{r}}$.

Prediction accuracy is measured in terms of MSPE. So the main question of interest is how the MSPE coming from the SPF fares relative to the MSPE of our two preferred naïve benchmarks: DRW+ and the RW+ . To evaluate forecast accuracy under MSPE, we focus on the difference


$$\Delta MSPE_{h}=E{\left[\overset{B}{\underset{t^{+}}{e}}(h)\right]}^{\text{2}}-E{\left[\overset{SPF}{\underset{t^{+}}{e}}(h)\right]}^{\text{2}}$$

Where $E{\left[\overset{B}{\underset{t^{+}}{e}}(h)\right]}^{\text{2}}$ represents the MSPE of a naïve benchmark (either DRW+ or RW+). We consider the following hypotheses


$$\begin{array}{cc} & {\text{H}}_{\text{0}}:\Delta MSPE_{h}\leq \text{0}\\ & {\text{H}}_{\text{A}}:\Delta {MSPE}_{h}>\text{0} \end{array}$$

Rejection of the null hypothesis implies that SPF forecasts outperform the corresponding benchmark at a statistically significant level. For inference, we apply a one-sided DMW test using HAC standard errors according to [26–27]. Tables 6 report results relative to the DRW+ , whereas Table 9 shows the results relative to the RW+ . While Table 6 focuses on our entire sample period, Tables 7 and 8 focus on the first and second subsample of interest, respectively. Entries in the tables show Root Mean Squared Prediction Error (RMSPE) ratios between SPF and our benchmarks. Ratios below 1 favor survey-based forecasts. Tables also display the t-statistic and p-value of the DMW test.

**Table 6:** Forecast accuracy of survey-based forecasts relative to the DRW+ at several forecasting horizons h (measured in months). April 2012 – April 2024 window.

	h = 1			h = 2			h = 3		
	RMSPE Ratio	DMW	p-value	RMSPE Ratio	DMW	p-value	RMSPE Ratio	DMW	p-value
**SPF2**	1.0819	−1.3041	0.9039	1.0644	−1.5499	0.9394	1.0442	−1.4956	0.9326
**SPF11**	1.3831	−2.7146	0.9967	1.2397	−2.3154	0.9897	1.1391	−1.6581	0.9513
**SPF23**	1.7457	−3.0201	0.9987	1.4613	−2.6299	0.9957	1.2723	−2.0580	0.9802
	**h = 6**			**h = 9**			**h = 11**		
	**RMSPE Ratio**	**DMW**	**p-value**	**RMSPE Ratio**	**DMW**	**p-value**	**RMSPE Ratio**	**DMW**	**p-value**
**SPF2**	1.0115	−0.4790	0.6840	**0.9964**	0.1720	0.4317	**0.9972**	0.1464	0.4418
**SPF11**	1.0246	−0.3833	0.6492	**0.9823**	0.3680	0.3371	**0.9872**	0.2612	0.3970
**SPF23**	1.0758	−0.8277	0.7961	1.0035	−0.0492	0.5196	1.0040	−0.0616	0.4806
	**h = 12**			**h = 18**			**h = 24**		
	**RMSPE Ratio**	**DMW**	**p-value**	**RMSPE Ratio**	**DMW**	**p-value**	**RMSPE Ratio**	**DMW**	**p-value**
**SPF2**	**0.9993**	0.0379	0.4849	1.0060	−0.3311	0.6297	1.0038	−0.3230	0.6266
**SPF11**	**0.9911**	0.1905	0.4245	1.0005	−0.0117	0.5047	**0.9919**	0.2644	0.3957
**SPF23**	1.0041	−0.0675	0.5269	1.0119	−0.2319	0.5917	**0.9967**	0.0723	0.4712

DMW test is constructed with HAC standard errors according to [26,27]. RMSPEs lower than 1 favor survey-based forecasts. * Significance at 10%, ** significance at 5%, ***significance at 1%.

**Table 7:** Forecast accuracy of survey-based forecasts relative to the DRW+ at several forecasting horizons h (measured in months). April 2012 – May 2018 window.

	h = 1			h = 2			h = 3		
	RMSPE Ratio	t	p-value	RMSPE Ratio	t	p-value	RMSPE Ratio	t	p-value
**SPF2**	1.0348	−0.5689	0.7153	**0.9660**	0.8421	0.1999	0.9583*	1.3090	0.0952
**SPF11**	1.1508	−1.6022	0.9454	**0.9755**	0.3592	0.3597	**0.9216**	1.2582	0.1042
**SPF23**	1.3303	−2.2502	0.9878	1.0535	−0.5091	0.6947	**0.9821**	0.1920	0.4239
	**h = 6**			**h = 9**			**h = 11**		
	**RMSPE Ratio**	**t**	**p-value**	**RMSPE Ratio**	**t**	**p-value**	**RMSPE Ratio**	**t**	**p-value**
**SPF2**	**0.9700**	1.2767	0.1009	**0.9837**	0.9310	0.1759	**0.9847**	0.8683	0.1926
**SPF11**	0.9160*	1.4108	0.0792	0.9125**	1.8666	0.0310	0.9192**	1.7201	0.0427
**SPF23**	**0.9320**	0.9026	0.1834	0.8990**	1.6996	0.0446	0.9017**	1.6564	0.0488
	**h = 12**			**h = 18**			**h = 24**		
	**RMSPE Ratio**	**t**	**p-value**	**RMSPE Ratio**	**t**	**p-value**	**RMSPE Ratio**	**t**	**p-value**
**SPF2**	**0.9822**	1.0342	0.1507	**0.9866**	0.8351	0.2018	0.9843*	1.4613	0.0720
**SPF11**	0.9212**	1.6929	0.0452	0.9256**	1.9380	0.0263	0.9280***	2.8310	0.0023
**SPF23**	0.9025*	1.6348	0.0510	0.9000**	1.9760	0.0241	0.8946***	2.8093	0.0025

DMW test is constructed with HAC standard errors according to [26–27]. RMSPEs lower than 1 favor survey-based forecasts. * Significance at 10%, ** significance at 5%, ***significance at 1%.

**Table 8:** Forecast accuracy of survey-based forecasts relative to the DRW+ at several forecasting horizons h (measured in months). June 2018 – April 2024 window.

	h = 1			h = 2			h = 3		
	RMSPE Ratio	t	p-value	RMSPE Ratio	t	p-value	RMSPE Ratio	t	p-value
**SPF2**	1.1038	−1.2222	0.8892	1.1233	−2.2394	0.9874	1.0912	−2.5497	0.9946
**SPF11**	1.4822	−2.9222	0.9983	1.3835	−3.2332	0.9994	1.2488	−2.4428	0.9927
**SPF23**	1.9140	−3.3549	0.9996	1.6724	−3.3264	0.9996	1.4145	−2.5477	0.9946
	**h = 6**			**h = 9**			**h = 11**		
	**RMSPE Ratio**	**t**	**p-value**	**RMSPE Ratio**	**t**	**p-value**	**RMSPE Ratio**	**t**	**p-value**
**SPF2**	1.0296	−0.9226	0.8219	1.0031	−0.1099	0.5438	1.0050	−0.1827	0.5725
**SPF11**	1.0701	−0.8643	0.8063	1.0177	−0.2561	0.6011	1.0281	−0.4338	0.6678
**SPF23**	1.1348	−1.1697	0.8789	1.0552	−0.5930	0.7234	1.0640	−0.7531	0.7743
	**h = 12**			**h = 18**			**h = 24**		
	**RMSPE Ratio**	**t**	**p-value**	**RMSPE Ratio**	**t**	**p-value**	**RMSPE Ratio**	**t**	**p-value**
**SPF2**	1.0108	−0.4023	0.6563	1.0270	−0.9569	0.8307	1.0309	−1.6641	0.9520
**SPF11**	1.0361	−0.5970	0.7248	1.0771	−1.5932	0.9444	1.0759	−2.4540	0.9929
**SPF23**	1.0678	−0.8665	0.8069	1.1226	−2.1556	0.9844	1.1255	−2.7590	0.9971

DMW test is constructed with HAC standard errors according to [26,27]. RMSPEs lower than 1 favor survey-based forecasts. * Significance at 10%, ** significance at 5%, ***significance at 1%.

**Table 9:** Forecast accuracy of survey-based forecasts relative to the RW+ at several forecasting horizons h (measured in months). June 2018 – April 2024 window.

	h = 1			h = 2			h = 3		
	RMSPE ratio	t	p-value	RMSPE ratio	t	p-value	RMSPE ratio	t	p-value
**SPF2**	1.1017	−1.1322	0.8712	**1.1350**	−2.0375	0.9792	**1.1112**	−1.8963	0.9710
**SPF11**	**1.4793**	−2.8604	0.9979	**1.3979**	−3.0406	0.9988	**1.2717**	−2.1304	0.9834
**SPF23**	**1.9103**	−3.3210	0.9996	**1.6898**	−3.2195	0.9994	**1.4404**	−2.3502	0.9906
	**h = 6**			**h = 9**			**h = 11**		
	**RMSPE ratio**	**t**	**p-value**	**RMSPE ratio**	**t**	**p-value**	**RMSPE ratio**	**t**	**p-value**
**SPF2**	1.0659	−0.8182	0.7934	1.0788	−0.7862	0.7841	1.1168	−1.0220	0.8466
**SPF11**	1.1077	−0.8105	0.7912	1.0944	−0.6515	0.7426	1.1425	−0.9145	0.8198
**SPF23**	1.1747	−1.0495	0.8530	1.1348	−0.7980	0.7876	1.1823	−0.9848	0.8509
	**h = 12**			**h = 18**			**h = 24**		
	**RMSPE ratio**	**t**	**p-value**	**RMSPE ratio**	**t**	**p-value**	**RMSPE ratio**	**t**	**p-value**
**SPF2**	1.1288	−1.1140	0.8674	1.1683	−1.1104	0.8666	1.0711	−0.3679	0.6435
**SPF11**	1.1570	−1.0322	0.8490	1.2253	−1.4053	0.9200	1.1179	−0.6210	0.7327
**SPF23**	1.1924	−1.1406	0.8730	**1.2770**	−1.7374	0.9588	1.1694	−0.8971	0.8152

DMW test is constructed with HAC standard errors according to [26,27]. RMSPEs lower than 1 favor survey-based forecasts. * Significance at 10%, ** significance at 5%, ***significance at 1%.

An analysis of the full sample results in Table 6 reveals that the SPF does not outperform the DRW+ in terms of accuracy. Although SPF2 and SPF11 exhibit reductions in RMSPE for medium-term horizons, these improvements are fairly small and not statistically significant according to the DMW test.

Tables 7 and 8 break down the results from Table 6 across the two subsample periods of interest. The analysis reveals that the relative predictive performance of the SPF fluctuates over time: Table 7 shows that the SPF outperforms the DRW+ at several horizons before May 2018, while Table 8 presents the opposite scenario, where the SPF is consistently outperformed by the DRW+ from June 2018 onward. The differences between Tables 7 and 8 are striking. For example, in Table 7, most of the RMSPE ratios are below 1 (23 out of 27), with 13 of them statistically significant at the 10% level. In stark contrast, all RMSPE ratios in Table 8 exceed 1, consistently favoring the DRW+ .

Finally, the results in Table 9 align with those in Table 8. Recall that Table 9 compares the SPF to the RW+ , which, for most forecasting horizons, serves as a more competitive benchmark than the DRW+ . As expected, most RMSPE ratios in Table 9 are higher than those in Table 8, with all exceeding 1, indicating that the SPF is consistently outperformed by the RW+ during the final subsample period. In summary, the results from Table 7, covering the period prior to May 2018, are largely consistent with those reported in [13]. However, the findings from June 2018 onward, presented in Tables 8 and 9, reveal a significant shift in the SPF’s predictive accuracy relative to the standard benchmarks. This change can primarily be attributed to the SPF’s consistent underestimation of the substantial depreciation of the Chilean Peso in the latter half of the sample period, a topic we will explore in more detail later.

#### 3.2.2. Mean directional accuracy.

Mean Directional Accuracy (MDA) is an alternative metric for evaluating the accuracy of a series of forecasts. Essentially, it assesses how often the direction of change—whether the exchange rate increases or decreases—is correctly predicted for a given forecast horizon. MDA is particularly relevant in our context because many economic and financial decisions depend primarily on anticipating the sign of exchange-rate movements—whether the currency will appreciate or depreciate—rather than the exact magnitude of those movements. For example, hedging strategies, carry-trade positions, and policy decisions often hinge on correctly forecasting the direction of returns. Importantly, MDA provides information that is distinct from MSPE and correlation. The MSPE is driven by the magnitude of forecast errors and may be dominated by large but infrequent deviations, while correlation measures only linear comovement between forecasts and realizations. By contrast, the MDA isolates the ability of a model to correctly anticipate the direction of change. It is therefore possible for two forecasts to have similar MSPEs or correlations but exhibit very different directional performance.

A crystal-clear case illustrating the need for MDA is the zero forecast. It is widely used as a benchmark in exchange-rate forecast evaluations because it delivers a low MSPE. However, its correlation with the target variable is not defined, since the zero forecast has zero variance, and moreover, it is absolutely independent of the variable being forecast. Most importantly, the zero forecast has no ability whatsoever to anticipate the direction of future exchange-rate movements. This makes it an ideal example showing that magnitude-based metrics alone cannot capture all dimensions of forecast usefulness.

To evaluate MDA, we construct the following “Hit Rate” statistic.


$$HR_{t^{+},h}=\left\{ \begin{array}{c} @l\text{1}\;if\;\left(s_{t+h}-s_{t^{+}}\right)\cdot \left(\overset{SPF}{\underset{t^{+}}{s}}-s_{t^{+}}\right)\;>\text{0}\\ \text{0}\;if\;\left(s_{t+h}-s_{t^{+}}\right)\cdot \left(\overset{SPF}{\underset{t^{+}}{s}}-s_{t^{+}}\right)\;\leq \;\text{0} \end{array}\right.\;$$ (3)

Then our hypotheses are


$$H_{\text{0}}:E\left[HR_{t^{+},h}\right]\leq \text{0}.\text{5},\;H_{A}:E\left[HR_{t^{+},h}\right]>\text{0}.\text{5}$$

In this case, we test whether the SPF outperforms a pure luck benchmark, implicitly defined as a forecast that predicts the direction of future movements in the Chilean Peso with a 50% probability of success. To evaluate this, we apply a one-sided Diebold-Mariano-West (DMW) test, using HAC standard errors as in [26–27].

Table 10 presents the results for the full sample period, while Tables 11 and 12 provide a breakdown for the two subsample periods of interest. Table 11 covers April 2012 to May 2018, and Table 12 spans June 2018 to April 2024. Each table reports the actual MDA for each forecast horizon, with statistically significant results highlighted by stars. An analysis of the full sample results in Table 10 reveals that the SPF outperforms the pure luck benchmark in only three cases, and even then, solely at the 10% significance level. The average hit rate across all horizons in Table 10 is a modest 52.4%. Notably, SPF11 achieves the highest MDA at nearly every horizon except h = 9. The peak performance in Table 10 is a MDA of 59.3%, achieved by SPF11 when forecasting 11 months ahead. Nevertheless, this outcome is not statistically distinguishable from the pure luck benchmark, as indicated by the DMW test. Overall, the findings in Table 10 are disappointing, mirroring the lackluster MSPE results reported for the full sample in Table 2.

**Table 10:** Mean Directional Accuracy of the SPF at several horizons. April 2012 – April 2024.

Horizon (months)	1	2	3	6	9	11	12	18	24
**SPF2**	51.7	50.0	48.3	45.7	46.0	48.9	47.0	50.8	46.7
**SPF11**	55.9*	55.6*	56.6*	51.4	56.2	59.3	56.0	56.3	53.3
**SPF23**	55.2	54.9	54.5	49.3	52.6	54.1	53.7	53.1	50.0

Gaussian t-statistic test is constructed with HAC standard errors. * Significance at 10%, ** significance at 5%, ***significance at 1%.

**Table 11:** Mean directional accuracy of the SPF at several horizons. April 2012 – May 2018.

Horizon (months)	1	2	3	6	9	11	12	18	24
**SPF2**	56.8*	58.9**	55.6	56.5	54.5	59.4	57.1	52.6	56.9
**SPF11**	63.5***	63.0***	65.3***	56.5	62.1*	67.2**	63.5	68.4**	76.5***
**SPF23**	60.8**	60.3**	62.5**	53.6	59.1	60.9	60.3	64.9*	72.5***
									

Gaussian t-statistic test is constructed with HAC standard errors. * Significance at 10%, ** significance at 5%, ***significance at 1%.

**Table 12:** Mean directional accuracy of the SPF at several horizons. June 2018 – April 2024.

Horizon (months)	1	2	3	6	9	11	12	18	24
**SPF2**	46.5	40.8	40.8	35.2	38.0	39.4	38.0	49.3	39.4
**SPF11**	47.9	47.9	47.9	46.5	50.7	52.1	49.3	46.5	36.6
**SPF23**	49.3	49.3	46.5	45.1	46.5	47.9	47.9	43.7	33.8

Gaussian t-statistic test is constructed with HAC standard errors. * Significance at 10%, ** significance at 5%, ***significance at 1%.

Tables 11 and 12 provide a detailed breakdown of the results from Table 10 across the two subsample periods. They reveal that the MDA of the SPF exhibits significant variation over time. For instance, Table 11 shows a strong behavior of the survey during our first subsample period. In particular, the SPF outperforms the pure luck benchmark at multiple horizons. Specifically, all entries in Table 11 exceed 50%, with 14 of them showing that the superiority over the benchmark is statistically significant. The average hit rate across Table 11 is an impressive 61.1%. In sharp contrast, Table 12 displays much weaker behavior, with only two entries displaying numbers above 50% and none achieving statistical significance. The average hit rate in Table 12 is a disappointing 44.5%, showing the stark difference in SPF behavior between the two periods.

Results based on MDA are largely consistent with those obtained from MSPE. In both cases, the SPF’s predictive performance varies over time: it outperforms our preferred naïve benchmark in the first subsample, but performs notably worse in the second subsample. In the next subsection, we will explore whether this pattern holds when evaluating predictive ability through correlations.

#### 3.2.3. Forecast evaluation based on correlations with the target variable.

Thus far we have evaluated the SPF’s ability to predict Chilean Peso returns by comparing its accuracy against conventional benchmarks. This approach, frequently employed in the exchange rate literature, has been used extensively in studies such as [1,5,10,13], among others. However, [15] argue that this intuitive and commonly applied methodology may not fully capture the true essence of predictability. As suggested by various authors, predictability fundamentally reflects a form of dependence between the target variable and past events or variables (As mentioned by [16] in p.657, *“The extent of a series predictability depends on how much information the past conveys regarding future values of this series”*.). This dependence can translate into greater predictive accuracy if forecasts are efficient in the [19] sense or exhibit only mild inefficiencies. As shown in [18], there are cases where a target variable exhibits a strong positive correlation with a forecast, even when the forecast is outperformed, based on accuracy metrics, by naïve and independent benchmarks. This distinction is at the core of their argument that predictability and forecast accuracy are separate concepts. To evaluate the former, they propose analyzing the correlation between the forecast and the target variable. Evidence of predictability arises when this correlation is positive or exceeds the correlation of a benchmark forecast with the target variable.

Tables 13-15 present the correlations between the three survey-based forecasts and Chilean Peso returns, defined as usual by the expression:

**Table 13:** Correlation between CLP+ and SPF at several horizons. April 2012 – April 2024 window.

	h = 1 month			h = 2			h = 3		
	Correlation	t	p-value	Correlation	t	p-value	Correlation	t	p-value
**SPF2**	0.1764**	1.7395	0.0410	0.1037	1.0561	0.1455	0.0739	1.0212	0.1536
**SPF11**	0.2003**	2.0695	0.0192	0.1542**	1.8737	0.0305	0.1497**	1.7534	0.0398
**SPF23**	0.1763**	1.8472	0.0324	0.1608**	1.8862	0.0296	0.1768**	1.7498	0.0401
	**h = 6**			**h = 9**			**h = 11**		
	**Correlation**	**t**	**p-value**	**Correlation**	**t**	**p-value**	**Correlation**	**t**	**p-value**
**SPF2**	0.0859	0.7726	0.2199	0.1275	0.9226	0.1781	0.1134	0.8036	0.2108
**SPF11**	0.2106*	1.3355	0.0909	0.2889*	1.5482	0.0608	0.2692*	1.3904	0.0822
**SPF23**	0.2526*	1.5545	0.0600	0.3473**	1.9942	0.0231	0.3449**	1.9737	0.0242
	**h = 12**			**h = 18**			**h = 24**		
	**Correlation**	**t**	**p-value**	**Correlation**	**t**	**p-value**	**Correlation**	**t**	**p-value**
**SPF2**	0.0997	0.6846	0.2468	0.0531	0.2804	0.3896	0.0205	0.1400	0.4443
**SPF11**	0.2565*	1.3279	0.0921	0.2300	1.1201	0.1313	0.2304*	1.3174	0.0939
**SPF23**	0.3473**	2.0259	0.0214	0.3429**	1.9300	0.0268	0.3624**	2.2732	0.0115

t statistics constructed with HAC standard errors. * Significance at 10%, ** significance at 5%, ***significance at 1%.

**Table 15:** Correlation between CLP+ and SPF at several horizons. June 2018 – April 2024 window.

	h = 1 month			h = 2			h = 3		
	Correlation	t	p-value	Correlation	t	p-value	Correlation	t	p-value
**SPF2**	0.1899	1.1218	0.1310	0.0759	0.4611	0.3223	0.0559	0.4751	0.3174
**SPF11**	0.2500*	1.5165	0.0647	0.1875**	1.9061	0.0283	0.2064**	2.0324	0.0211
**SPF23**	0.2132*	1.3922	0.0819	0.2043*	1.6318	0.0514	0.2889***	2.6676	0.0038
	**h = 6**			**h = 9**			**h = 11**		
	**Correlation**	**t**	**p-value**	**Correlation**	**t**	**p-value**	**Correlation**	**t**	**p-value**
**SPF2**	0.1433	0.9369	0.1744	0.2699**	2.0798	0.0188	0.2523**	1.8518	0.0320
**SPF11**	0.3817***	2.5107	0.0060	0.5179***	4.1195	0.0000	0.4744***	3.2767	0.0005
**SPF23**	0.4727***	3.3148	0.0005	0.6004***	4.4505	0.0000	0.5750***	3.8640	0.0001
	**h = 12**			**h = 18**			**h = 24**		
	**Correlation**	**t**	**p-value**	**Correlation**	**t**	**p-value**	**Correlation**	**t**	**p-value**
**SPF2**	0.2140*	1.4950	0.0675	0.1456	0.8051	0.2104	−0.0404	−0.2540	0.6003
**SPF11**	0.4438***	3.1471	0.0008	0.2767*	1.3497	0.0886	0.1003	0.4326	0.3327
**SPF23**	0.5669***	4.0874	0.0000	0.4160**	2.1338	0.0164	0.2126	0.8704	0.1920

t statistics constructed with HAC standard errors. * significance at 10%, ** significance at 5%, ***significance at 1%. Correlations are actual correlations values.


$$r_{t^{+},t+h}=s_{t+h}-s_{t^{+}}$$

where $s_{t^{+}}$represents the log-exchange rate observed immediately before the survey’s release and $s_{t+h}$ is the log-exchange rate *h* periods ahead. Table 13 summarizes results for the full sample period, whereas Table 14 focuses on the first subsample of interest and Table 15 examines the last subsample. For inference, we consider the following simple regression:

**Table 14:** Correlation between CLP+ and SPF at several horizons. April 2012 – May 2018 window.

	h = 1 month			h = 2			h = 3		
	Correlation	t	p-value	Correlation	t	p-value	Correlation	t	p-value
**SPF2**	0.1654*	1.2930	0.0980	0.2017**	1.6484	0.0496	0.2080*	1.5589	0.0595
**SPF11**	0.2308**	1.7844	0.0372	0.2730**	1.9239	0.0272	0.3333**	2.1874	0.0144
**SPF23**	0.2250*	1.5077	0.0658	0.2663*	1.6064	0.0541	0.2789*	1.5167	0.0647
	**h = 6**			**h = 9**			**h = 11**		
	**Correlation**	**t**	**p-value**	**Correlation**	**t**	**p-value**	**Correlation**	**t**	**p-value**
**SPF2**	0.1171	1.1895	0.1171	−0.0208	−0.1652	0.5656	−0.0486	−0.3652	0.6425
**SPF11**	0.2629**	1.9757	0.0241	0.2531**	1.6859	0.0459	0.2107*	1.2951	0.0977
**SPF23**	0.2538*	1.5635	0.0590	0.3051**	2.0026	0.0226	0.2925**	1.8462	0.0324
	**h = 12**			**h = 18**			**h = 24**		
	**Correlation**	**t**	**p-value**	**Correlation**	**t**	**p-value**	**Correlation**	**t**	**p-value**
**SPF2**	−0.0393	−0.3035	0.6192	−0.1209	−0.6674	0.7478	0.0508	0.2939	0.3844
**SPF11**	0.1865	1.0924	0.1373	0.2287	1.0557	0.1456	0.4459***	2.4802	0.0066
**SPF23**	0.2823**	1.7106	0.0436	0.3673**	2.1990	0.0139	0.6143***	4.8709	0.0000

t statistics constructed with HAC standard errors. * significance at 10%, ** significance at 5%, ***significance at 1%. Correlations are actual correlations values.


$$r_{t^{+},t+h}=\alpha +\beta \cdot \overset{SPF}{\underset{t^{+}}{r}}+\varepsilon_{t^{+},t+h}$$ (4)

In this framework,


$$\beta =\frac{Cov(\overset{SPF}{\underset{t^{+}}{r}};r_{t^{+},t+h})}{Var(\overset{SPF+}{\underset{t^{+}}{r}})}$$

We test the following hypotheses:


$$H_{\text{0}}:\beta =\text{0},\;H_{A}:\beta >\text{0}$$

$H_{\text{0}}$ posits that the predictor and target variables are uncorrelated, implying linear independence. To test this, we use a usual t-statistic constructed with HAC standard errors according to [26,27]. Tables 13–15 present not only the correlation coefficients between the three survey-based forecasts and Chilean Peso returns, but also the corresponding t-statistics and p-values for testing the null hypothesis β=0.

The results across the three samples are striking. Correlations are mostly positive, relatively high, and statistically significant at several horizons in all three tables. At a 90% confidence level, Table 13 shows that the null hypothesis is rejected in 16 out of 27 cases, while both Tables 14 and 15 show 19 rejections out of 27 cases. The average correlation across all entries in Table 13 is 0.198, with the comparable figures for Tables 14 and 15 being 0.216 and 0.284, respectively. Unlike the findings from MSPE and MDA analyses, correlation results show a relatively stable behavior across both subsamples of interest. Additionally, there is evidence that the SPF performs best during the last subsample period, even though it was clearly outperformed by naïve benchmarks in terms of MSPE and MDA during the same period.

Table 16 presents results from a slightly different exercise, where we report the following correlation differential:


$$Corr\left(\overset{SPF}{\underset{t^{+}}{r}};r_{t^{+},t+h}\right)-Corr\left(\overset{RW+}{\underset{t^{+}}{r}}(h);r_{t^{+},t+h}\right)$$ (5)

In words, we are comparing the correlation of the SPF with the target variable with the correlation of the RW+ forecast with the same target variable. This comparison helps us to assess whether survey-based forecasts exhibit a stronger correlation with the target variable than the RW+ forecast. For inference, we use the asymptotically normal correlation-based test proposed by [28] to test the null hypothesis of a zero-correlation differential. This test evaluates the correlation of two competing forecasts Z and X with a target variable Y, all of them which are supposed to be stationary variables. This test requires the construction of the following vectors


$$W=W_{t}=(\begin{array}{c} Z_{t}\\ X_{t}\\ Y_{t}\\ \overset{\text{2}}{\underset{t}{Z}}\\ \overset{\text{2}}{\underset{t}{X}}\\ Z_{t}Y_{t}\\ X_{t}Y_{t} \end{array})=(\begin{array}{c} Z\\ X\\ Y\\ Z^{\text{2}}\\ X^{\text{2}}\\ ZY\\ XY \end{array}),\text{ and }\tilde{W}=W-E(W)=(\begin{array}{c} Z-E(Z)\\ X-E(X)\\ Y-E(Y)\\ Z^{\text{2}}-E(Z^{\text{2}})\\ X^{\text{2}}-E(X^{\text{2}})\\ ZY-E(ZY)\\ XY-E(XY) \end{array})$$

Consider the following assumptions i), ii), iii) and iv):

i) The vector $\tilde{W}$ is strictly stationary with mixing coefficients α(l) such that, for some r>2, E${\left\|\tilde{W}\right\|}^{r}<\infty$ and $\sum_{l=\text{1}}^{\infty }\alpha (l{)}^{\text{1}-\frac{\text{2}}{r}}<\infty$
ii) A strictly positive variance for Y, X, and Z.
iii) X and Z are considered as primitives (i.e., with no parameter uncertainty).
iv) Corr(Y,X) and Corr (Y,Z) are both strictly lower than 1.

Under these assumptions, [28] show that the following t-statistic


$$Correlation-t=\sqrt{T\overset{\text{2}}{\underset{y}{s}}}\;\left(\frac{r_{\text{z}}-r_{\text{x}}}{\sqrt{\hat{V}}}\right)$$

is asymptotically normal under the null hypothesis. Here $\hat{V}=\nabla {\hat{h}}^{\prime }\nabla {\hat{g}}^{\prime }[\sum_{j=-\infty }^{\infty }{\hat{\Omega }}_{\text{j}}]\;\nabla \hat{g}\nabla \hat{h}$, $r_{\text{z}}$ and ${\text{r}}_{\text{x}}$ represent the sample correlations of Z and X with Y, respectively. Besides $\overset{\text{2}}{\underset{y}{s}}$ is the sample variance of the target variable, T is the number of forecasts, $\nabla {\hat{h}}^{\prime }=[-\frac{\text{1}}{\text{2}}{(s}_{yz}/\;\overset{\text{3}}{\underset{z}{s}});\;\frac{\text{1}}{\text{2}}{(s}_{yx}/\overset{\text{3}}{\underset{x}{s}});\;\text{1}/s_{z};\;\text{1}/s_{x}]$, where $s_{z},\;s_{x},\;s_{yx}$ and $s_{yz}$ denote the sample standard deviations and covariances, respectively, and


$$\begin{array}{c} \nabla \hat{g}=(\begin{array}{cccc} -\text{2}\overset{―}{z} & \text{0} & -\overset{―}{y} & \text{0}\\ \text{0} & -\text{2}\overset{―}{x} & \text{0} & -\overset{―}{y}\\ \text{0} & \text{0} & -\overset{―}{z} & -\overset{―}{x}\\ \text{1} & \text{0} & \text{0} & \text{0}\\ \text{0} & \text{1} & \text{0} & \text{0}\\ \text{0} & \text{0} & \text{1} & \text{0}\\ \text{0} & \text{0} & \text{0} & \text{1} \end{array}),\; \end{array}$$

where $\overset{―}{z},\;\overset{―}{x}$ and $\overset{―}{y}$ represent the sample mean of Z, X, and Y respectively. Finally, $\Omega_{\text{j}}=$


$$\begin{array}{c} \mathrm{\backslash footnotesize}(\begin{array}{ccccccc} Cov(Z_{t},Z_{t-j}) & Cov(X_{t},Z_{t-j}) & Cov(Y_{t},Z_{t-j}) & Cov(\overset{\text{2}}{\underset{t}{Z}},Z_{t-j}) & Cov(\overset{\text{2}}{\underset{t}{X}},Z_{t-j}) & Cov(Z_{t}Y_{t},Z_{t-j}) & Cov(X_{t}Y_{t},Z_{t-j})\\ Cov(Z_{t},X_{t-j}) & Cov(X_{t},X_{t-j}) & Cov(Y_{t},X_{t-j}) & Cov(\overset{\text{2}}{\underset{t}{Z}},X_{t-j}) & Cov(\overset{\text{2}}{\underset{t}{X}},X_{t-j}) & Cov(Z_{t}Y_{t},X_{t-j}) & Cov(X_{t}Y_{t},X_{t-j})\\ Cov(Z_{t},Y_{t-j}) & Cov(X_{t},Y_{t-j}) & Cov(Y_{t},Y_{t-j}) & Cov(\overset{\text{2}}{\underset{t}{Z}},Y_{t-j}) & Cov(\overset{\text{2}}{\underset{t}{X}},Y_{t-j}) & Cov(Z_{t}Y_{t},Y_{t-j}) & Cov(X_{t}Y_{t},Y_{t-j})\\ Cov(Z_{t},\overset{\text{2}}{\underset{t-j}{Z}}) & Cov(X_{t},\overset{\text{2}}{\underset{t-j}{Z}}) & Cov(Y_{t},\overset{\text{2}}{\underset{t-j}{Z}}) & Cov(\overset{\text{2}}{\underset{t}{Z}},\overset{\text{2}}{\underset{t-j}{Z}}) & Cov(\overset{\text{2}}{\underset{t}{X}},\overset{\text{2}}{\underset{t-j}{Z}}) & Cov(Z_{t}Y_{t},\overset{\text{2}}{\underset{t-j}{Z}}) & Cov(X_{t}Y_{t},\overset{\text{2}}{\underset{t-j}{Z}})\\ Cov(Z_{t},\overset{\text{2}}{\underset{t-j}{X}}) & Cov(X_{t},\overset{\text{2}}{\underset{t-j}{X}}) & Cov(Y_{t},\overset{\text{2}}{\underset{t-j}{X}}) & Cov(\overset{\text{2}}{\underset{t}{Z}},\overset{\text{2}}{\underset{t-j}{X}}) & Cov(\overset{\text{2}}{\underset{t}{X}},\overset{\text{2}}{\underset{t-j}{X}}) & Cov(Z_{t}Y_{t},\overset{\text{2}}{\underset{t-j}{X}}) & Cov(X_{t}Y_{t},\overset{\text{2}}{\underset{t-j}{X}})\\ Cov(Z_{t},Z_{t-j}Y_{t-j}) & Cov(X_{t},Z_{t-j}Y_{t-j}) & Cov(Y_{t},Z_{t-j}Y_{t-j}) & Cov(\overset{\text{2}}{\underset{t}{Z}},Z_{t-j}Y_{t-j}) & Cov(\overset{\text{2}}{\underset{t}{X}},Z_{t-j}Y_{t-j}) & Cov(Z_{t}Y_{t},Z_{t-j}Y_{t-j}) & Cov(X_{t}Y_{t},Z_{t-j}Y_{t-j})\\ Cov(Z_{t},X_{t-j}Y_{t-j}) & Cov(X_{t},X_{t-j}Y_{t-j}) & Cov(Y_{t},X_{t-j}Y_{t-j}) & Cov(\overset{\text{2}}{\underset{t}{Z}},X_{t-j}Y_{t-j}) & Cov(\overset{\text{2}}{\underset{t}{X}},X_{t-j}Y_{t-j}) & Cov(Z_{t}Y_{t},X_{t-j}Y_{t-j}) & Cov(X_{t}Y_{t},X_{t-j}Y_{t-j}) \end{array}) \end{array}$$

We present results of this test for our second subsample of interest only. This is because the drift of the RW+ is estimated as a rolling average of the most recent observations available in the sample. Specifically, we use the first subsample to obtain the initial estimate of the drift, and then update this estimate in rolling windows of the same size (74 observations). Table 16 reports not only the correlation differential in (5) but also the corresponding t-statistics and p-values coming from the correlation-based test.

**Table 16:** Correlation Differences: RW+ vs SPF at several horizons. June 2018 – April 2024 window.

	h = 1 month			h = 2			h = 3		
	D(Correlation)	t	p-value	D(Correlation)	t	p-value	D(Correlation)	t	p-value
**SPF2**	0.3253**	2.0716	0.0192	0.2052	1.1083	0.1339	0.2772*	1.6031	0.0545
**SPF11**	0.3854***	2.4894	0.0064	0.3168**	1.6665	0.0478	0.4277***	3.1453	0.0008
**SPF23**	0.3487***	2.6955	0.0035	0.3336**	2.1934	0.0141	0.5102***	4.2015	0.0000
	**h = 6**			**h = 9**			**h = 11**		
	**D(Correlation)**	**t**	**p-value**	**D(Correlation)**	**t**	**p-value**	**D(Correlation)**	**t**	**p-value**
**SPF2**	0.4773***	2.4546	0.0071	0.4294**	1.9217	0.0273	0.3267	1.2608	0.1037
**SPF11**	0.7157***	3.3175	0.0005	0.6773***	2.3770	0.0087	0.5488**	1.6625	0.0482
**SPF23**	0.8067***	3.8530	0.0001	0.7599***	2.6056	0.0046	0.6494**	1.8482	0.0323
	**h = 12**			**h = 18**			**h = 24**		
	**D(Correlation)**	**t**	**p-value**	**D(Correlation)**	**t**	**p-value**	**D(Correlation)**	**t**	**p-value**
**SPF2**	0.2815	1.0570	0.1453	0.2346	0.6162	0.2689	0.1672	0.4383	0.3306
**SPF11**	0.5113*	1.5150	0.0649	0.3657	0.8987	0.1844	0.3079	0.7332	0.2317
**SPF23**	0.6344**	1.7403	0.0409	0.5050	1.1975	0.1156	0.4202	1.0043	0.1576

t statistics constructed with HAC standard errors. * significance at 10%, ** significance at 5%, ***significance at 1%.

The overall pattern in Table 16 closely mirrors the results in Table 15. It shows that all correlation differentials are positive, indicating a clear advantage for the SPF over the RW+. Additionally, 18 entries in Table 16 are statistically significant in favor of the SPF. These findings further support and reinforce the conclusions drawn from Table 15.

In summary, we find that the relative forecast accuracy of the survey has declined in the second subsample. However, its correlation has improved at several horizons. Moreover, a close inspection of Table 8 vs Table 15 and of Table 9 vs Table 16 reveals that, in many cases, our naïve benchmarks outperform the SPF in terms of MSPE, while the SPF outperforms these naïve benchmarks in terms of correlations. This scenario is referred to as the MSPE Paradox by [18]. The paradox occurs when the forecast with the highest correlation with the target variable also exhibits the worst MSPE. As shown by [18], a key condition for the MSPE Paradox to arise is the inefficiency of some of the forecasts involved in the comparison. Consequently, our paradoxical results suggest that our survey-based forecasts might be inefficient, a topic we explore in the following section.

### 3.3. Efficiency analysis

The MSPE Paradox by [18] emerges if at least one of the forecasts involved in the comparison is inefficient, as defined by [19]. This implies that at least one forecast is either biased or exhibits a nonzero correlation with its own forecast error. The evidence of the MSPE Paradox presented in the previous subsection, coupled with the clear bias observed in the survey, as shown in Fig 2, leads us to further investigate the potential inefficiencies of the survey. We start by analyzing bias and then the correlation of survey-based forecasts with their own forecast errors.

#### 3.3.1. Bias in survey-based forecasts.

A forecast is deemed biased if the expected value of its forecast error deviates from zero. To test for this, we consider the simple regression specified in expression (6), regressing the forecast error on a constant. As is standard practice, we apply HAC standard errors following [26,27].


$$\overset{SPF}{\underset{t^{+}}{e}}\left(h\right)=\overset{SPF}{\underset{h}{\delta }}+\varepsilon_{t+h}\;$$ (6)

We test the following hypotheses


$$H_{0}:E\left[\overset{SPF}{\underset{t^{+}}{e}}\left(h\right)\right]=0,\;\;\;\;\;\;\;\;\;\;\;\;\;H_{A}:E\left[\overset{SPF}{\underset{t^{+}}{e}}\left(h\right)\right]\neq 0$$

Rejection of the null implies that the forecast is biased. Results are shown in Table 17 for the full sample, and in Tables 18 and 19 for the two subsamples of interest.

**Table 17:** Bias in survey-based forecasts. April 2012 – April 2024 window.

	h = 1 month			h = 2			h = 3		
	E(e)	t	p-value	E(e)	t	p-value	E(e)	t	p-value
**SPF2**	0.5493*	1.7254	0.0845	1.0052**	1.9635	0.0496	1.4311**	1.9762	0.0481
**SPF11**	1.0760*	1.6764	0.0937	1.5069**	2.0058	0.0449	1.8981**	2.1134	0.0346
**SPF23**	1.8014**	2.0197	0.0434	2.2116**	2.2835	0.0224	2.5797**	2.3961	0.0166
	h = 6			h = 9			h = 11		
	E(e)	t	p-value	E(e)	t	p-value	E(e)	t	p-value
**SPF2**	2.7570**	2.1516	0.0314	4.1537**	2.3820	0.0172	5.0276**	2.545	0.0109
**SPF11**	3.1510**	2.3412	0.0192	4.4679**	2.5664	0.0103	5.3241***	2.7309	0.0063
**SPF23**	3.8075***	2.6661	0.0077	5.1170***	2.9176	0.0035	5.9645***	3.0962	0.0020
	h = 12			h = 18			h = 24		
	**E(e)**	**t**	**p-value**	**E(e)**	**t**	**p-value**	**E(e)**	**t**	**p-value**
**SPF2**	5.4573***	2.6307	0.0085	8.1074***	3.1716	0.0015	11.0806***	3.8395	0.0001
**SPF11**	5.7559***	2.8206	0.0048	8.3735***	3.3562	0.0008	11.1847***	3.9734	0.0001
**SPF23**	6.3964***	3.193	0.0014	9.0112***	3.7507	0.0002	11.7079***	4.3466	0.0000

E(e) denotes the expected value of survey-based forecast errors. * Significance at 10%, ** significance at 5%, ***significance at 1%. Forecast errors are scale up by 100.

**Table 18:** Bias in survey-based forecasts. April 2012 – May 2018 window.

	h = 1			h = 2			h = 3		
	E(e)	t	p-value	E(e)	t	p-value	E(e)	t	p-value
**SPF2**	−0.1052	−0.3355	0.7372	0.2296	0.4286	0.6682	0.4527	0.5735	0.5663
**SPF11**	−0.9361**	−2.4074	0.0161	−0.6126	−1.0521	0.2927	−0.3897	−0.4787	0.6322
**SPF23**	−1.0056*	−1.7173	0.0859	−0.6721	−0.8930	0.3718	−0.4387	−0.4583	0.6468
	**h = 6**			**h = 9**			**h = 11**		
	**E(e)**	**t**	**p-value**	**E(e)**	**t**	**p-value**	**E(e)**	**t**	**p-value**
**SPF2**	1.4193	0.8915	0.3727	2.7666	1.2345	0.2170	3.7950	1.4373	0.1506
**SPF11**	0.5877	0.3803	0.7037	1.9211	0.8870	0.3751	2.9354	1.1525	0.2491
**SPF23**	0.5719	0.3593	0.7194	1.9282	0.8998	0.3682	2.9430	1.1836	0.2366
	**h = 12**			**h = 18**			**h = 24**		
	**E(e)**	**t**	**p-value**	**E(e)**	**t**	**p-value**	**E(e)**	**t**	**p-value**
**SPF2**	4.4077	1.5654	0.1175	8.0035**	2.0522	0.0402	11.7305**	2.4029	0.0163
**SPF11**	3.5226	1.2955	0.1951	7.1299*	1.9030	0.057	10.8556**	2.3340	0.0196
**SPF23**	3.5184	1.3299	0.1835	7.0861**	1.9735	0.0484	10.8209**	2.4865	0.0129

E(e) denotes the expected value of survey-based forecast errors. * Significance at 10%, ** significance at 5%, ***significance at 1%. Forecast errors are scale up by 100.

**Table 19:** Bias in survey-based forecasts. June 2018 – April 2024 window.

	h = 1 month			h = 2			h = 3		
	E(e)	t	p-value	E(e)	t	p-value	E(e)	t	p-value
**SPF2**	1.2315***	2.7433	0.0061	1.8026**	2.3465	0.019	2.4232**	2.1936	0.0283
**SPF11**	3.1731***	4.6827	0.0000	3.6862***	4.0804	0.0000	4.2182***	3.6408	0.0003
**SPF23**	4.7270***	5.5729	0.0000	5.1766***	5.1393	0.0000	5.6406***	4.7205	0.0000
	**h = 6**			**h = 9**			**h = 11**		
	**E(e)**	**t**	**p-value**	**E(e)**	**t**	**p-value**	**E(e)**	**t**	**p-value**
**SPF2**	4.0571**	2.1177	0.0342	5.4431**	2.2748	0.0229	6.1387**	2.4299	0.0151
**SPF11**	5.6420***	3.1130	0.0019	6.8354***	3.0804	0.0021	7.4772***	3.1776	0.0015
**SPF23**	6.9520***	4.0494	0.0001	8.0813***	3.9274	0.0001	8.6881***	4.0057	0.0001
	**h = 12**			**h = 18**			**h = 24**		
	**E(e)**	**t**	**p-value**	**E(e)**	**t**	**p-value**	**E(e)**	**t**	**p-value**
**SPF2**	6.3885**	2.5017	0.0124	8.1909***	3.2540	0.0011	10.6138***	4.6449	0.0000
**SPF11**	7.7375***	3.2353	0.0012	9.3719***	3.8456	0.0001	11.4212***	4.9592	0.0000
**SPF23**	8.9502***	4.0597	0.0000	10.5566***	4.6437	0.0000	12.3450***	5.4900	0.0000

E(e) denotes the expected value of survey-based forecast errors. * Significance at 10%, ** significance at 5%, ***significance at 1%. Forecast errors are scale up by 100.

Table 17 shows a positive and statistically significant bias in the SPF across all forecasting horizons. The first entry in Table 17 indicates a bias of 0.5493 for SPF2 when h = 1, meaning that the SPF2 underestimates the Chilean Peso by approximately 0.55% when forecasting one-month-ahead. The corresponding entry for SPF23, when forecasting 24 months ahead, is 11.7079. This means that SPF23 underestimates the Chilean Peso by approximately 12% when forecasting two-years-ahead. In contrast, when analyzing the sample from April 2012 to May 2018 in Table 18, the results differ considerably. A positive and statistically significant bias is observed only when forecasting 18 and 24 months ahead. At shorter horizons, however, the bias is either small or negative, and more importantly, not statistically significant. Table 19 completes the whole picture by presenting results for the subsample from June 2018 to April 2024. Similar to Table 17, Table 19 shows a positive and statistically significant bias across all entries. This bias is relatively small for SPF2 when forecasting one-month-ahead (approximately 1.2%) but becomes substantial at longer horizons, well above 10% when h = 24.

#### 3.3.2. Correlation of survey-based forecasts with forecast errors.

In this section, we examine another type of forecast inefficiency: a non-zero correlation between survey-based forecasts and their own forecast errors. We test this using the following regression:


$$\overset{SPF}{\underset{t^{+}}{e}}\left(h\right)=\overset{SPF}{\underset{h}{\text{a}}}+\overset{SPF}{\underset{h}{\beta }}\overset{SPF}{\underset{t^{+}}{r}}+w_{t^{+},t+h}$$ (7)

As usual, we use HAC standard errors according to [26,27]. Our hypotheses are as follows:


$$\begin{array}{cc} & H_{\text{0}}:\;\overset{SPF}{\underset{h}{\beta }}=\;0\\ \;\; & H_{A}:\;\overset{SPF}{\underset{h}{\beta }}\neq 0 \end{array}$$

The null hypothesis posits that our survey-based forecasts are linearly independent of their own forecast errors. Although we have built three tables covering our results for the full sample and both subsamples of interest, in Table 20 we will only show results for the second subsample, from June 2018 to April 2024, for the sake of brevity. The rest of our results are available upon request. Table 20 shows statistically significant and negative results for $\overset{SPF}{\underset{h}{\beta }}$ at short horizons, specifically for h = 1, 2 and 3. There are also some positive and statistically significant results for $\overset{SPF}{\underset{h}{\beta }}$ at h = 9. For the rest of the forecasting horizons, we cannot reject the null of zero correlation between survey–based forecasts and their own forecast errors. Results for the full sample and the first subsample of interest are similar, with statistically significant results occurring only for a handful of forecasting horizons.

**Table 20:** Correlation of survey-based forecasts with forecast errors. June 2018 – April 2024 window.

	h = 1 month			h = 2			h = 3		
	Beta	t	p-value	Beta	t	p-value	Beta	t	p-value
**SPF2**	−0.6535**	−2.1155	0.0344	−0.8273**	−2.2084	0.0272	−0.8366**	−2.4324	0.0150
**SPF11**	−0.7132***	−3.7713	0.0002	−0.7298***	−5.1489	0.0000	−0.6145***	−3.2396	0.0012
**SPF23**	−0.8039***	−5.7084	0.0000	−0.7636***	−5.2711	0.0000	−0.5660***	−3.4789	0.0005
	**h = 6**			**h = 9**			**h = 11**		
	**Beta**	**t**	**p-value**	**Beta**	**t**	**p-value**	**Beta**	**t**	**p-value**
**SPF2**	−0.3326	−0.4669	0.6406	0.5460	0.7345	0.4626	0.4477	0.5727	0.5669
**SPF11**	0.1256	0.2802	0.7794	0.9234**	1.9777	0.048	0.7462	1.4002	0.1615
**SPF23**	0.0848	0.2591	0.7955	0.6503*	1.7538	0.0795	0.5675	1.3989	0.1619
	**h = 12**			**h = 18**			**h = 24**		
	**Beta**	**t**	**p-value**	**Beta**	**t**	**p-value**	**Beta**	**t**	**p-value**
**SPF2**	0.2582	0.3068	0.759	−0.1678	−0.1623	0.8711	−1.2026	−1.5081	0.1315
**SPF11**	0.6759	1.2692	0.2044	−0.0604	−0.0868	0.9308	−0.6922	−0.9728	0.3307
**SPF23**	0.5818	1.5033	0.1328	0.0599	0.1206	0.904	−0.5059	−0.8911	0.3729

t statistics constructed with HAC standard errors. * significance at 10%, ** significance at 5%, ***significance at 1%.

In summary, we have found that survey-based forecasts are inefficient, particularly in consistently underpredicting the Chilean Peso in our final subsample of interest. In the next subsection, we will explore if these inefficiencies can be used to generate new and more accurate forecasts.

### 3.4. Forecast optimization

In this subsection, we present an adjustment to the SPF forecasts aimed at improving their accuracy. We begin by estimating the inefficiencies in the original SPF forecasts and then subtract these from the original values to derive the final Adjusted SPF forecasts.

We start by estimating regression (7) for our first subsample of interest. Using the initial estimates of $\overset{SPF}{\underset{h}{\text{a}}}\text{and }\overset{SPF}{\underset{h}{\beta }}$ we compute the following adjusted forecast $A\overset{SPF}{\underset{t^{+}}{r}}\left(h\right)$:


$$A\overset{SPF}{\underset{t^{+}}{r}}\left(h\right)=\overset{SPF}{\underset{h}{\text{a}}}+\left(1+\overset{SPF}{\underset{h}{\beta }}\right)\overset{SPF}{\underset{t^{+}}{r}}$$ (8)

If either $\overset{SPF}{\underset{h}{\text{a}}}$ or $\overset{SPF}{\underset{h}{\beta }}$ is statistically and economically significant, the adjusted forecast $A\overset{SPF}{\underset{t^{+}}{r}}\left(h\right)$ is expected to show greater accuracy relative to the original forecast. Using the initial estimates of $\overset{SPF}{\underset{h}{\text{a}}}\overset{SPF}{\underset{h}{\text{and}\beta }}$, we generate a new forecast for the first relevant observation in our second subsample of interest. For example, when forecasting one-month-ahead, the first observation corresponds to June 2018. To generate an adjusted forecast for the second relevant observation, we re-estimate expression (7) using the most recent 74 observations, updating the estimates of $\overset{SPF}{\underset{h}{\text{a}}}\text{and }\overset{SPF}{\underset{h}{\beta }}$. With these new estimates, we construct the adjusted forecast for the second observation, corresponding to July 2018 when forecasting one-month-ahead. This process is repeated iteratively using rolling windows of 74 observations to generate adjusted forecasts for all subsequent observations. By employing this strategy, we simulate a real-time out-of-sample experiment. If the rolling estimates from expression (7) effectively capture stable inefficiencies, the adjusted forecasts should demonstrate higher accuracy.

Table 21 confirms this result, presenting RMSPE ratios between the Adjusted SPF and the raw SPF, where figures lower than 1 favor the former. Almost all the ratios in Table 21 are well below one, with only a couple of exceptions when forecasting 24 months ahead. The average ratio across all horizons is 0.874, favoring the Adjusted SPF. Improvements in forecast accuracy are all statistically significant at the short horizons of 1,2 and 3 months. At longer horizons there is only one entry showing statistically significant results for SPF23 when h = 18. The lowest ratio in Table 21, an impressive 0.53 for SPF23 at h = 1, highlights the substantial effectiveness of the adjustment. Despite this significant improvement, Table 22 brings us back to reality by presenting RMSPE ratios between the Adjusted SPF and our toughest benchmark, the RW+. All figures in Table 22 are above 1, favoring the benchmark. While the Adjusted SPF shows notable improvements, it still falls short of outperforming the RW+ in terms of MSPE.

**Table 21:** Forecast accuracy of the Adjusted SPF relative to the SPF without adjustment. June 2018 – April 2024 window.

	h = 1 month			h = 2			h = 3		
	RMSPE Ratio	t	p-value	RMSPE Ratio	t	p-value	RMSPE Ratio	t	p-value
**SPF2**	0.912*	1.446	0.074	0.907**	1.731	0.042	0.919**	1.719	0.043
**SPF11**	0.684***	2.948	0.002	0.742***	2.949	0.002	0.824**	1.980	0.024
**SPF23**	0.531***	3.364	0.000	0.609***	3.220	0.001	0.716***	2.396	0.008
	**h = 6**			**h = 9**			**h = 11**		
	**RMSPE Ratio**	**t**	**p-value**	**RMSPE Ratio**	**t**	**p-value**	**RMSPE Ratio**	**t**	**p-value**
**SPF2**	0.961	0.550	0.291	0.950	0.456	0.324	0.908	0.748	0.227
**SPF11**	0.966	0.341	0.366	0.965	0.262	0.397	0.920	0.513	0.304
**SPF23**	0.907	0.691	0.245	0.908	0.682	0.247	0.873	0.848	0.198
	**h = 12**			**h = 18**			**h = 24**		
	**RMSPE Ratio**	**t**	**p-value**	**RMSPE Ratio**	**t**	**p-value**	**RMSPE Ratio**	**t**	**p-value**
**SPF2**	0.904	0.800	0.212	0.906	0.576	0.282	0.991	0.051	0.480
**SPF11**	0.912	0.609	0.271	0.936	0.465	0.321	1.087	−0.578	0.718
**SPF23**	0.869	0.962	0.168	0.839*	1.397	0.081	0.941	0.380	0.352

DMW test is constructed with HAC standard errors. * Significance at 10%, ** significance at 5%, ***significance at 1%. Ratios below 1 favor the Adjusted SPF.

**Table 22:** Forecast accuracy of the Adjusted SPF relative to the RW+ . June 2018 – April 2024 window.

	h = 1 month			h = 2			h = 3		
	RMSPE Ratio	t	p-value	RMSPE Ratio	t	p-value	RMSPE Ratio	t	p-value
**SPF2**	1.005	−0.197	0.578	1.030	−2.251	0.988	1.021	−1.760	0.961
**SPF11**	1.011	−0.503	0.692	1.038	−1.782	0.963	1.047	−1.689	0.954
**SPF23**	1.015	−0.756	0.775	1.029	−1.547	0.939	1.032	−1.109	0.866
	**h = 6**			**h = 9**			**h = 11**		
	**RMSPE Ratio**	**t**	**p-value**	**RMSPE Ratio**	**t**	**p-value**	**RMSPE Ratio**	**t**	**p-value**
**SPF2**	1.024	−2.232	0.987	1.025	−1.052	0.854	1.014	−0.450	0.674
**SPF11**	1.070	−1.862	0.969	1.056	−1.346	0.911	1.051	−0.758	0.776
**SPF23**	1.065	−1.918	0.972	1.030	−0.470	0.681	1.032	−0.399	0.655
	**h = 12**			**h = 18**			**h = 24**		
	**RMSPE Ratio**	**t**	**p-value**	**RMSPE Ratio**	**t**	**p-value**	**RMSPE Ratio**	**t**	**p-value**
**SPF2**	1.020	−0.682	0.752	1.058	−0.690	0.755	1.061	−3.248	0.999
**SPF11**	1.056	−0.742	0.771	1.147	−1.964	0.975	1.215	−3.299	1.000
**SPF23**	1.036	−0.418	0.662	1.072	−1.374	0.915	1.101	−1.493	0.932

DMW test is constructed with HAC standard errors. * significance at 10%, ** significance at 5%, ***significance at 1%. Ratios below 1 favor the Adjusted SPF.

Table 23 presents more encouraging results, showing the MDA of the Adjusted SPF across all horizons. In contrast to the less favorable results for the raw SPF in Table 12, we observe consistent improvements for horizons longer than 2 months.

**Table 23:** Mean directional accuracy of the adjusted SPF. June 2018– April 2024.

Horizon (months)	1	2	3	6	9	11	12	18	24
**SPF2**	43.7	50.7	56.3	63.3*	70.4**	70.4**	67.6*	73.2***	71.8**
**SPF11**	45.1	46.5	46.5	57.7	66.2*	70.4**	70.4**	77.5***	59.2
**SPF23**	47.9	47.9	46.5	59.2	66.2**	71.8**	70.4**	78.9***	64.8

Gaussian t-statistics are constructed with HAC standard errors. * Significance at 10%, ** significance at 5%, ***significance at 1%.

Furthermore, values greater than 50% are achieved when forecasting 6 months ahead or longer. For horizons ranging from 9 to 18 months, the results are statistically and economically superior to the pure luck benchmark. The adjustment proves effective in forecasting the direction of change of the Chilean Peso, particularly at medium to long-term horizons. The highest average hit rate is attained with SPF2 when forecasting 18 months ahead, with a notable figure of 78.9%.

We also explored an alternative strategy to address potential inefficiencies in the survey, by assessing whether forecast errors at a given horizon can be partially explained by information from forecasts at other horizons as well. In particular, we examined whether the forecast errors of SPF2, SPF11, and SPF23 can be systematically related to the information contained in the remaining forecasts. To this end, we employed the following regression framework:


$$\overset{SPF}{\underset{t^{+}}{e}}\left(h\right)=\overset{SPF}{\underset{h}{\text{a}}}+\overset{\left(1\right)}{\underset{h}{\beta }}\overset{SPF2}{\underset{t^{+}}{r}}+\overset{\left(2\right)}{\underset{h}{\beta }}\overset{SPF11}{\underset{t^{+}}{r}}+\overset{\left(3\right)}{\underset{h}{\beta }}\overset{SPF23}{\underset{t^{+}}{r}}+u_{t^{+},t+h}$$ (9)

where $\overset{\text{SPF}}{\underset{{\text{t}}^{+}}{\text{e}}}\left(\text{h}\right)\text{\textbackslash{} }$ represents a generic h-step-ahead forecast error that can be generated either by SPF2, SPF11 or SPF23. This expression allows for a more general adjustment of the raw forecasts coming from the survey. Equation (9) is estimated using the LASSO method, which enables variable selection and reduces estimation noise in settings with multicollinearity and limited sample sizes, relative to standard OLS.

To our general disappointment, this strategy does not yield significant improvements over the simpler adjustment approach based on equations (7) and (8). Table 24 illustrates this by reporting RMSPE ratios between the LASSO-adjusted SPF and our most challenging benchmark, the RW+. As in Table 22, all ratios in Table 24 are above 1, indicating superior performance by the benchmark. In fact, the RMSPE ratios in Table 24 are slightly higher than those in Table 22, suggesting a marginal advantage for the simpler OLS-based adjustment.

**Table 24:** Forecast accuracy of the LASSO-Adjusted-SPF relative to the RW+. June 2018 – April 2024 window.

	h = 1 month			h = 2			h = 3		
	**RMSPE Ratio**	**t**	**p-value**	**RMSPE Ratio**	**t**	**p-value**	**RMSPE Ratio**	**t**	**p-value**
**SPF2**	1.0552	−1.1516	0.8753	1.086	−1.916	0.972	1.081	−2.294	0.989
**SPF11**	1.0366	−0.7810	0.7826	1.089	−2.237	0.987	1.094	−2.037	0.979
**SPF23**	1.0306	−0.8240	0.7950	1.046	−1.477	0.930	1.054	−1.080	0.860
	**h = 6**			**h = 9**			**h = 11**		
	**RMSPE Ratio**	**t**	**p-value**	**RMSPE Ratio**	**t**	**p-value**	**RMSPE Ratio**	**t**	**p-value**
**SPF2**	1.034	−0.893	0.814	1.014	−0.350	0.637	1.036	−0.741	0.771
**SPF11**	1.065	−1.016	0.845	1.016	−0.210	0.583	1.047	−0.597	0.725
**SPF23**	1.064	−0.851	0.803	1.005	−0.058	0.523	1.020	−0.206	0.582
	**h = 12**			**h = 18**			**h = 24**		
	**RMSPE Ratio**	**t**	**p-value**	**RMSPE Ratio**	**t**	**p-value**	**RMSPE Ratio**	**t**	**p-value**
**SPF2**	1.034	−0.661	0.746	1.070	−1.602	0.282	1.063	−2.390	0.992
**SPF11**	1.055	−0.727	0.766	1.117	−1.553	0.940	1.099	−2.063	0.980
**SPF23**	1.023	−0.249	0.598	1.100	−1.231	0.891	1.087	−1.500	0.933

DMW test is constructed with HAC standard errors. * Significance at 10%, ** significance at 5%, ***significance at 1%. Ratios below 1 favor the Adjusted SPF.

We also evaluated directional accuracy (MDA) using the LASSO-based adjustment obtaining slightly less favorable results than those presented in Table 19. For example, the average hit rate in Table 19 is 61.5%, whereas the corresponding figure in the table based on the LASSO adjustment is only 56.6%. For brevity, this additional table is not included here but is available upon request. As with the RMSPE results, the more complex LASSO-based adjustment does not appear to offer a compelling improvement over the simpler OLS-based alternative.

In conclusion, either the OLS or the LASSO Adjusted SPF exhibits considerable improvements in forecast accuracy compared to the original, unadjusted SPF. While these improvements are not enough to outperform our preferred benchmark in terms of MSPE, they are more than enough to outperform the pure luck benchmark in terms of MDA at medium horizons.

We also applied the Elastic Net to equation (9). This method combines the LASSO penalty, which performs variable selection with the Ridge penalty, which stabilizes coefficient estimates under multicollinearity. We assign an equal weight of 0.5 to both penalties and use a cross-validation procedure (customized to time-series) to choose the optimal regularization parameter. The results are qualitatively similar as those obtained with the LASSO method. Although the adjusted forecast clearly outperforms the SPF forecast (Table 25) it fails reducing the forecast errors of the RW+ (Table 26).

**Table 25:** Forecast accuracy of the Elastic Net-Adjusted-SPF relative to the SPF without adjustment. June 2018 – April 2024 window.

	h = 1 month			h = 2			h = 3		
	**RMSPE Ratio**	**t**	**p-value**	**RMSPE Ratio**	**t**	**p-value**	**RMSPE Ratio**	**t**	**p-value**
**SPF2**	0.937*	1.608	0.054	0.933*	1.424	0.074	0.951*	1.444	0.074
**SPF11**	0.682***	4.441	0.000	0.756***	3.512	0.000	0.835***	2.951	0.002
**SPF23**	0.533***	5.355	0.000	0.618***	4.762	0.000	0.718***	4.154	0.000
	**h = 6**			**h = 9**			**h = 11**		
	**RMSPE Ratio**	**t**	**p-value**	**RMSPE Ratio**	**t**	**p-value**	**RMSPE Ratio**	**t**	**p-value**
**SPF2**	0.941**	1.645	0.049	0.909**	2.020	0.021	0.894**	1.957	0.025
**SPF11**	0.884***	2.429	0.007	0.888**	2.279	0.011	0.851***	2.353	0.009
**SPF23**	0.818***	3.255	0.001	0.806***	3.570	0.000	0.800***	3.030	0.001
	**h = 12**			**h = 18**			**h = 24**		
	**RMSPE Ratio**	**t**	**p-value**	**RMSPE Ratio**	**t**	**p-value**	**RMSPE Ratio**	**t**	**p-value**
**SPF2**	0.859***	2.589	0.004	0.810***	3.016	0.001	0.753***	2.979	0.000
**SPF11**	0.815***	2.938	0.001	0.774***	3.567	0.000	0.734***	3.528	0.000
**SPF23**	0.784***	3.413	0.000	0.738***	4.375	0.000	0.665***	5.305	0.000

Notes: DMW test is constructed with HAC standard errors. * Significance at 10%, ** significance at 5%, ***significance at 1%. Ratios below 1 favor the Adjusted SPF.

**Table 26:** Forecast accuracy of the Elastic Net-Adjusted-SPF relative to the RW+ . June 2018 – April 2024 window.

	h = 1 month			h = 2			h = 3		
	RMSPE Ratio	t	p-value	RMSPE Ratio	t	p-value	RMSPE Ratio	t	p-value
**SPF2**	1.028	−1.410	0.920	1.044	−2.482	0.993	1.044	−2.536	0.994
**SPF11**	1.020	−0.956	0.830	1.040	−1.759	0.960	1.053	−2.346	0.990
**SPF23**	1.028	−1.748	0.960	1.030	−1.661	0.951	1.028	−1.338	0.909
	**h = 6**			**h = 9**			**h = 11**		
	**RMSPE Ratio**	**t**	**p-value**	**RMSPE Ratio**	**t**	**p-value**	**RMSPE Ratio**	**t**	**p-value**
**SPF2**	1.031	−1.388	0.917	0.999	0.038	0.484	1.021	−0.611	0.729
**SPF11**	1.009	−0.342	0.633	0.989	0.315	0.376	0.995	0.133	0.447
**SPF23**	0.992	0.225	0.410	0.929**	1.671	0.047	0.966	0.914	0.180
	**h = 12**			**h = 18**			**h = 24**		
	**RMSPE Ratio**	**t**	**p-value**	**RMSPE Ratio**	**t**	**p-value**	**RMSPE Ratio**	**t**	**p-value**
**SPF2**	0.988	0.341	0.367	1.058	−1.123	0.869	1.216	−2.278	0.989
**SPF11**	0.962	0.956	0.170	1.061	−1.187	0.882	1.238	−2.436	0.993
**SPF23**	0.954	1.134	0.128	1.054	−1.041	0.851	1.175	−1.938	0.974

Notes: DMW test is constructed with HAC standard errors. * Significance at 10%, ** significance at 5%, ***significance at 1%. Ratios below 1 favor the Adjusted SPF.

## 4. Discussion

Our results prompt several interesting discussions, one of which involves the selection of benchmarks for evaluating exchange rate predictability. While [10] identifies the driftless random walk (DRW) as the toughest benchmark to beat, our findings demonstrate that the random walk with drift (RW+) outperforms both the DRW and the DRW+ when forecasting the Chilean peso over long horizons. This outcome can be attributed to the significant depreciation of the CLP during the sample period. Thus, similar results may arise for other currencies that exhibit sustained trending behavior, independently of their specific institutional or macroeconomic characteristics. This observation follows from a statistical mechanism implied by equation (1): when an exchange rate displays a persistent trend—whether an extended depreciation or appreciation—the estimated drift is non-zero. In such cases, imposing a zero-drift restriction introduces systematic forecast errors, whereas allowing for a non-zero drift (as in the RW or RW+) captures the deterministic component of the trend and therefore tends to improve forecast performance.

The second discussion relates to the accuracy of the SPF in predicting the CLP. Similar to the findings reported by [13], the SPF’s MSPE—now measured from the day of respondent’s response—is statistically lower than that of the benchmark prior to May 2018 at several forecasting horizons. However, the survey’s performance deteriorates significantly after this date, both in terms of MSPE and Mean Directional Accuracy. This decline may be attributed to the SPF’s persistent downward bias observed after May 2018, suggesting that experts struggled to anticipate the magnitude of the depreciation that followed in the years thereafter.

Third, the relatively stable, positive and significant correlation between the CLP and the SPF across the entire sample—spanning multiple horizons—clearly suggests predictability. However, when coupled with the SPF’s underperformance in terms of accuracy, this serves as a clear illustration of the MSPE Paradox discussed by [18]. This paradox suggests that a strong dependence between the target variable and its predictor does not necessarily lead to a reduction in MSPE when compared to an independent benchmark, such as the DRW. Our findings show that the MSPE Paradox is not merely a theoretical artifact but a tangible phenomenon affecting Chilean peso forecasts produced by the SPF. This opens an intriguing avenue for future research: investigating whether this paradox also manifests in surveys for other exchange rates or in broader forecasting contexts.

Finally, we have applied a simple yet effective method for adjusting the inefficiencies of the SPF, which improves its accuracy in terms of both MSPE and MDA. However, it does not consistently outperform the RW+ in terms of MSPE. Thus, a key challenge for future research is to explore ways of transforming forecast inefficiencies into higher accuracy, with the goal of outperforming traditional benchmarks.

As mentioned in the introduction, accurate forecasts of the Chilean peso are not only valuable for practitioners and households interested in the future value of the CLP per se. It also has broader implications. As demonstrated by [6,20,29], commodity-currencies like the CLP have predictive power for metal and fuel prices. Therefore, our Adjusted SPF can also be of use to experts in those markets.

An important avenue for future research would be to assess the robustness of our inference results in settings where comparisons were conducted without explicitly adjusting for the pre-selection of the best-performing methods. In particular, this issue is most relevant for the evaluation of the optimal benchmark identified in the first part of the paper, where the benchmark-selection step precedes the formal tests of predictive ability. Incorporating procedures that account for such pre-selection would allow us to determine whether the benchmark’s apparent superiority remains statistically valid once this additional source of uncertainty is taken into consideration.
